# The Prion Protein Controls Polysialylation of Neural Cell Adhesion Molecule 1 during Cellular Morphogenesis

**DOI:** 10.1371/journal.pone.0133741

**Published:** 2015-08-19

**Authors:** Mohadeseh Mehrabian, Dylan Brethour, Hansen Wang, Zhengrui Xi, Ekaterina Rogaeva, Gerold Schmitt-Ulms

**Affiliations:** 1 Tanz Centre for Research in Neurodegenerative Diseases, University of Toronto, Toronto, Ontario, Canada; 2 Department of Laboratory Medicine & Pathobiology, University of Toronto, Toronto, Ontario, Canada; 3 Department of Medicine, University of Toronto, Toronto, Ontario, Canada; University of Maryland School of Medicine, UNITED STATES

## Abstract

Despite its multi-faceted role in neurodegenerative diseases, the physiological function of the prion protein (PrP) has remained elusive. On the basis of its evolutionary relationship to ZIP metal ion transporters, we considered that PrP may contribute to the morphogenetic reprogramming of cells underlying epithelial-to-mesenchymal transitions (EMT). Consistent with this hypothesis, PrP transcription increased more than tenfold during EMT, and stable PrP-deficient cells failed to complete EMT in a mammalian cell model. A global comparative proteomics analysis identified the neural cell adhesion molecule 1 (NCAM1) as a candidate mediator of this impairment, which led to the observation that PrP-deficient cells fail to undergo NCAM1 polysialylation during EMT. Surprisingly, this defect was caused by a perturbed transcription of the polysialyltransferase ST8SIA2 gene. Proteomics data pointed toward β-catenin as a transcriptional regulator affected in PrP-deficient cells. Indeed, pharmacological blockade or siRNA-based knockdown of β-catenin mimicked PrP-deficiency in regards to NCAM1 polysialylation. Our data established the existence of a PrP-ST8SIA2-NCAM signaling loop, merged two mature fields of investigation and offer a simple model for explaining phenotypes linked to PrP.

## Introduction

Ever since evidence mounted that the prion protein is the causative agent underlying prion diseases [1], yet is widely expressed in healthy vertebrate cells of diverse lineages, scientists have sought to uncover the physiological role of this protein [2]. The cellular prion protein (PrP^C^) has been tied to diverse cellular activities ranging from cell adhesion to ion transport, neuritogenesis, modulation of electrophysiological currents and circadian regulation (reviewed in [3,4,5,6]) but the molecular mechanism of its proposed involvement in these and other activities has remained largely enigmatic. It is a challenge to identify a prevailing theme in this body of literature, a reality reflected in the widely held view that the role of this protein is complex, multifaceted and context-dependent. The limitations of our current understanding of the physiological role of PrP were further accentuated when it was proposed that PrP^C^ plays a critical role in a central signaling pathway in Alzheimer’s disease (AD) [7]. It is to be expected that efforts to intervene with PrP’s pathogenic role in neurodegenerative diseases would benefit from a thorough understanding of both the cellular programs that control its expression and the principal signaling pathways that may contribute to toxic signals emanating from PrP.

When considering the relative merits of alternative approaches for determining the physiological role of a given protein, three methods stand out, namely, one may choose to infer its function from the function of its molecular interactors [8,9], deduce it from the function of its closest evolutionary relative [10], or characterize the phenotypic consequences of disease causing mutations, including gene knockouts [11].

Several studies uncovered molecular interactors of PrP^C^ or proteins residing in its spatial proximity [12,13,14]. Cumulatively, these data suggest the prion protein is enriched in lipid raft membrane domains and surrounded by several cell adhesion molecules, including NCAM1 and integrin or non-integrin laminin receptors. NCAM1 seems particularly enriched amongst proteins residing in proximity to PrP and this next-neighbor relationship can be captured by mild *in vivo* formaldehyde crosslinking of cultured cells [13,15] or brain tissue [14]. The physiological significance of this molecular proximity has remained largely unclear. One scenario sees PrP cooperate with NCAM1 in its recruitment to lipid rafts and signaling to the tyrosine kinase FYN [16]. In separate research, NCAM1 has been identified as a mediator of epithelial-to-mesenchymal transition (EMT), a morphogenetic reprogramming of epithelial cells that causes them to break out of the epithelial cell layer and acquire fibroblastoid characteristics [17].

Recently, several lines of evidence converged to reveal that the prion founder gene was derived from an ancient ZIP (Zrt-, Irt-like protein) metal ion transporter gene [18] through a genomic rearrangement that involved the genomic insertion of a spliced ZIP mRNA intermediate [19]. Several vertebrate genomes are known to code for more than a dozen ZIP transporter paralogs, with ZIP5, ZIP6 and ZIP10 being most similar to PrP on the basis of their PrP-like ectodomains, which share up to 30% sequence identity with PrP sequences in a subset of species [18]. The morpholino-mediated knockdown of ZIP6 was observed to cause a gastrulation arrest phenotype during zebrafish embryogenesis, characterized by a failure of cells to complete EMT and migrate along the anterior-posterior axis [20]. Similar phenotypes of defective EMT-like cellular migration programs, leading to perturbed gonad and trachea formation, have been reported to occur in fruit flies deficient for a ZIP ortholog [21,22].

To date, the most striking observation related to PrP-deficiency was generated by morpholino-mediated knockdown of a PrP ortholog in zebrafish (PrP-1) [23,24]. The study recorded a gastrulation arrest phenotype during embryonic development that is highly reminiscent of the one observed in ZIP6-deficient embryos [25].

On the basis of these three strands of observations, we hypothesized that PrP might play a role in EMT and wondered if the protein affects the execution of this program also in mammalian cells [26]. Here we document that PrP is more than tenfold upregulated during EMT and cells deficient for PrP fail to complete EMT. Whereas wild-type cells undergo NCAM1 polysialylation during EMT, stable PrP-deficient cells fail to do so due to an impairment in expressing sialyltransferase ST8SIA2, a critical mediator of this key post-translational modification. We then show that β-catenin plays a role in the transcriptional regulation of ST8SIA2 and propose a novel transcriptional feedback loop. Finally we discuss the significance of this work for understanding the function of PrP, highlighting conspicuous similarities in previous reports on PrP and PSA-NCAM1.

## Results

### PrP contributes to morphogenetic reprogramming of cells during EMT

**Fig 1:**
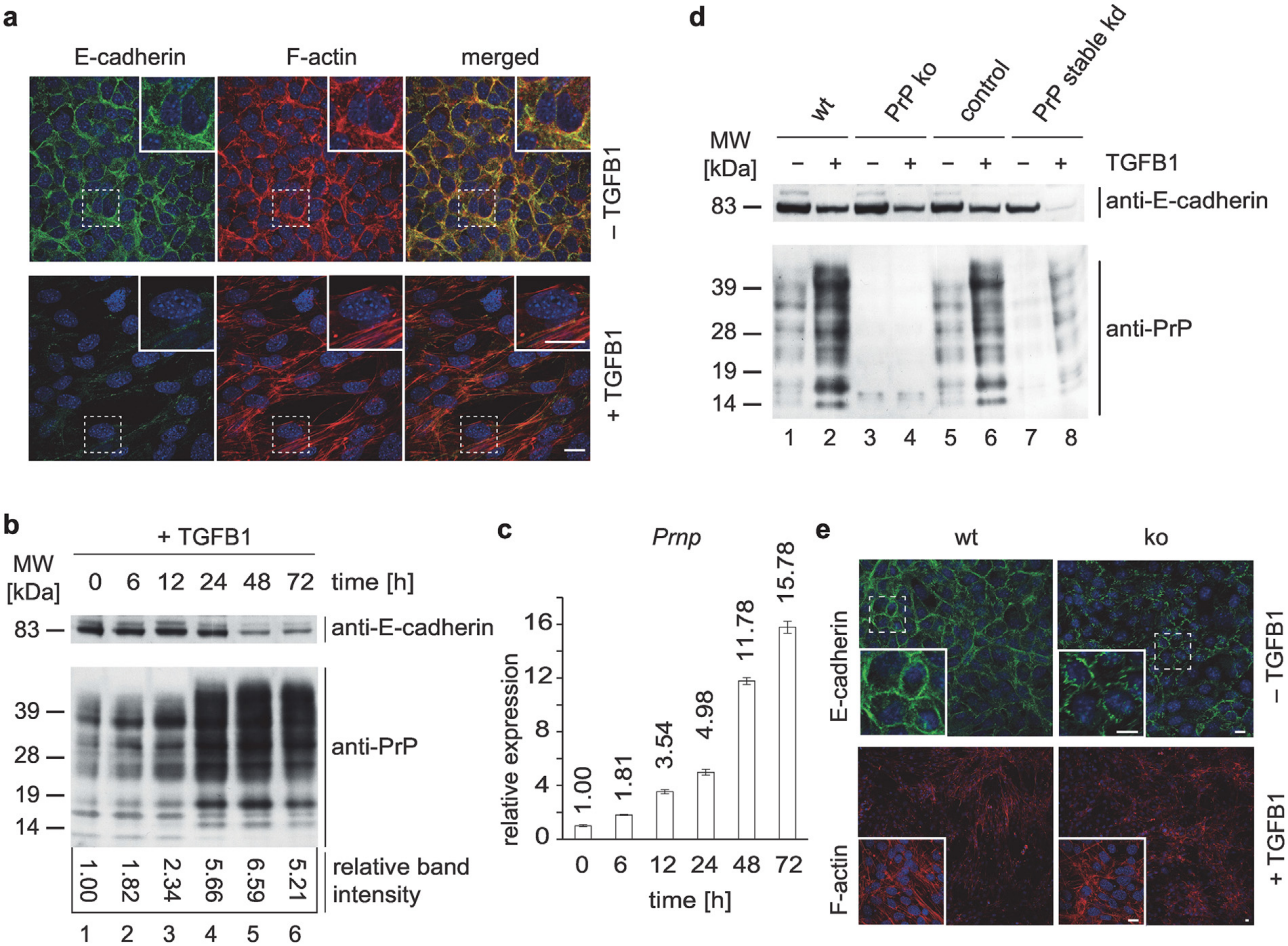
PrP^C^ expression is transcriptionally upregulated during EMT. (a) Double-immunofluorescence analyses of NMuMG cells before and after 48 h exposure to TGFB1, depicting the changes to cell shape and actin cytoskeleton that accompany EMT in this cell model. (b) Western blot analysis of E-cadherin and PrP^C^ protein levels in NMuMG cell extracts during 72 h of exposure to TGFB1. (c) Profound upregulation of *Prnp* gene transcription accounts for increased PrP^C^ protein levels during EMT based on a time-course RT-PCR analysis of PrP transcripts in NMuMG cells following addition of TGFB1 to the cell culture medium. (d) Comparison of E-cadherin and PrP protein levels in wt NMuMG cells and PrP-deficient derivative cell clones obtained by CRISRP-Cas9-based PrP knockout or stable shRNA-based kd. The ‘negative control’ represents a cell clone which had been subjected to identical CRISPR-Cas9-based *Prnp* knockout procedures but did not result in a PrP knockout. (e) Immunofluorescence analysis of E-cadherin and F-actin in wt or PrP-deficient cells before and after TGFB1 exposure. Disorganized E-cadherin distribution at cell-cell junctions and failure of PrP-deficient cells to exhibit directional alignment following TGFB1 exposure.

Mouse mammary gland epithelial cells (NMuMG) [27] are a widely used model for studying EMT because this cell line responds robustly to transforming growth factor beta 1 (TGFB1) exposure with morphogenetic reprogramming that bears all hallmarks of EMT [28]. Consistent with prior reports, persistent exposure of NMuMG cells to TGFB1 stimulated the expected transdifferentiation, with cadherin levels declining as cells lose their adherens junctions and acquire a fibroblastoid morphology (Fig 1a). As early as 6 h into the time-course of TGFB1 exposure, PrP protein levels increased and continued to climb until 48 hrs, when a peak level was reached that exceeded levels in mock-treated cells more than 6-fold (Fig 1b).

To investigate if EMT-associated changes in PrP levels had arisen from increases in transcriptional activity of the *Prnp* gene or changes to PrP translation or turnover, real-time PCR (RT-PCR) analyses were undertaken. A robust accumulation of *Prnp*-transcripts was observed during the time-course of TGFB1 treatment, with fifteenfold higher *Prnp* expression after three days of treatment compared to transcript levels in untreated cells (Fig 1c).

To address if transcriptional activation of PrP is essential for EMT or represents merely a correlative phenomenon, we next compared the execution of this program in NMuMG wild-type (wt) cells or derivative clones whose levels of PrP expression were diminished by CRISPR/Cas9 knockout (ko) technology or stable knockdown (kd) of PrP transcripts (Fig 1d) [29]. When these alternative PrP-deficiency models were monitored before and after exposure to TGFB1, several observations were made: (i) PrP-deficient NMuMG cells exhibited defects in cell-cell contacts even prior to EMT-induction that manifested in a perturbed distribution of E-cadherin at the plasma membrane, characterized by a zigzag, non-continuous appearance of its signals along the plasma membrane. (ii) Upon addition of TGFB1, PrP-deficient cells acquired a less pronounced fibroblastoid phenotype than wt cells. More specifically, whereas wt cells acquired a spindle-like shape and seemed to align, with groups of cells sharing the same longitudinal axis direction, PrP-deficient cells were exhibiting these properties to a lesser degree (Fig 1e).

### Comparative proteome analyses identify NCAM1 as a candidate for mediating PrP’s influence on EMT

**Fig 2:**
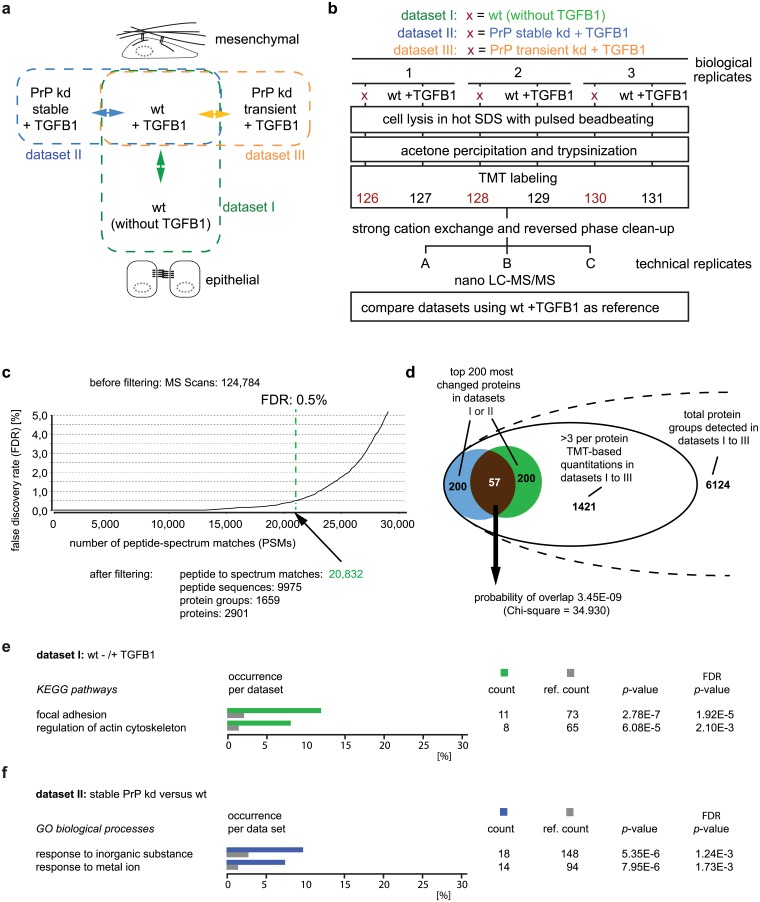
Quantitative mass spectrometry identifies perturbed ‘response to metal ions’ and EMT markers, including NCAM1, affected in PrP-deficient cells. (a) Design of quantitative global proteome comparisons giving rise to datasets I to III. (b) Workflow of global proteome analyses conducted by comparative mass spectrometry. Note that this workflow was executed 3 times to generated datasets I to III, with the ‘x’ being replaced by the respective condition specified at the top of this panel. To facilitate comparison of datasets, the three experiments differed in the biological samples which were labeled with even-numbered TMT reagents. All three datasets shared the use of wt NMuMG cell extracts following 48 h TGFB1 exposure as reference samples labeled with odd-numbered TMT reagents. (c) Example graph depicting post-acquisition filtering of datasets and benchmarks of mass spectrometry analysis (shown for dataset I). (d) Profound overlap amongst top 200 proteins whose levels are most changed during EMT or following stable PrP kd. (e) Exposure of NMuMG cells to TGFB1 causes changes to proteins whose KEGG annotations identify them as players in pathways that contribute to ‘focal adhesion’ formation and ‘actin cytoskeleton regulation’. (f) Direct comparison of global proteomes of wt and stable PrP kd NMuMG cells following TGFB1 exposure identifies highly significant perturbations in biological processes with ‘response to inorganic substance’ and ‘response to metal ions’ GO annotations.

To identify proteins that contribute to EMT in NMuMG cells and are affected in PrP-deficient clones, we next conducted three comparative global proteome analyses. Whereas the first analysis was intended to reveal proteins whose levels are altered in wt cells during EMT (dataset I), the second and third analyses addressed the question which of the proteins, present in TGFB1- treated NMuMG cells, are affected following stable (dataset II) or transient (dataset III) knockdown of PrP transcripts (Fig 2a). For rapid cell lysis and destruction of nucleic acids, equal amounts of cell pellets were lysed in hot SDS and subjected to beadbeating. To facilitate the direct comparison of PrP wt and kd samples and their biological replicates, trypsinized peptides were isobarically labeled with tandem mass tags (TMT) [30] in a six-plex format and analyzed by mass spectrometry. Because three biological replicates of TGFB1-treated wt NMuMG cells were common to all three analyses conducted, their global proteome served as a reference against which three biological replicates of each of the following conditions were compared: (1) wt NMuMG cells, without TGFB1, (2) TGFB1-treated stable or (3) TGFB1-treated transient PrP kd NMuMG cells (the rational for including this dataset will become apparent later) (Fig 2b). Each of the three comparative analyses gave rise to more than 20,000 peptide-to-spectrum matches (PSMs). For example, the direct comparison of wt cells ± TGFB1 yielded 20,832 PSMs that could be assigned to >9,975 peptides and 1,659 protein groups, meeting a stringent 0.5% false discovery rate (FDR) filter (Fig 2c). The ‘protein group’ term is used to indicate that for more than a third of PSMs unequivocal assignments to proteins cannot be made due to the existence of separate database entries for protein isoforms or close homologs that may share amino acid sequences. The downstream comparisons of data sets were restricted to 1,421 proteins for which quantitation data from a minimum of three spectra covering the low-mass region (comprising TMT signature ions) were available (Fig 2d, S1 Table). A Kruskal-Wallis H test applied to protein levels observed within biological replicates revealed non-significant differences (e.g., *p* = 0.934), indicative of a high level of data reproducibility.

**Fig 3:**
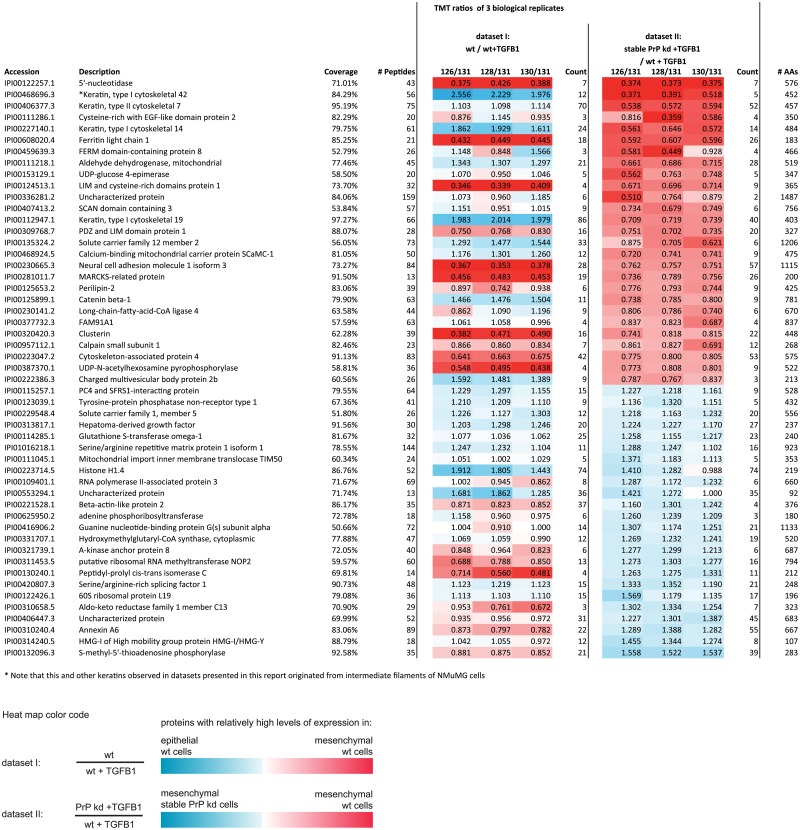
PrP-deficiency affects expression of a subset of proteins undergoing pronounced expression levels changes during EMT. List of proteins exhibiting >20% level differences in comparison of global proteomes of TGFB1-treated stable PrP kd versus wt NMuMG cells (dataset II). Coverage: percentages of primary structure of covered by peptide-to-spectrum matches; # Peptides: number of peptides matched to a given protein entry (note that instances of the same peptide being identified with different modifications counted separately in this tally); Count: number of TMT signature ion distributions, which passed stringent filtering criteria and were used for relative quantitation. Please see S1 Table for complete list of proteins identified, including control samples, confidence scores and statistical measures.

Consistent with expectations for cells undergoing EMT, a subsequent KEGG pathway analysis pointed toward a significant occurrence of proteins with known roles in focal adhesion formation and regulation of the actin cytoskeleton amongst the list of proteins whose levels changed most profoundly in response to TGFB1 exposure (dataset I, Fig 2e, S2 Table). We next determined the overlap amongst the lists of proteins whose levels were most affected by TGFB1 induction or stable PrP kd (dataset II, S3 Table). Strikingly, this analysis registered an overlap of 57 amongst the 200 proteins most affected by these two independent manipulations (Fig 2d, S4 Table), corresponding to a probability of 3.45E-09 (Chi-square = 34.930) if this overlap was due to chance alone. Although this established a highly significant connection of PrP to proteins whose levels changed in EMT, the direction of protein levels changes in cells which underwent EMT (dataset I) was not correlated to the protein level changes caused by stable PrP-deficiency in TGFB1 treated cells (dataset II) (Spearman correlation; *ƿ* = 0.419). This result suggested that stable PrP kd does not impair EMT by shifting the cellular proteome towards a more epithelial or mesenchymal phenotype but rather disturbs the natural balance of EMT-related proteins. Interestingly, a gene ontology (GO) analysis of proteins, whose levels were most affected by stable PrP kd, revealed a significant enrichment of entries annotated to participate in the biological process of ‘response to inorganic substances’ and its GO child term ‘response to metals’ (Fig 2f). Close inspection of the datasets flagged NCAM1 as a protein of interest, because it underwent profound induction during EMT, its levels differed by more than 20% in a comparison of TGFB1-treated wt and stable PrP kd cells (Fig 3 **and panel a in** S1 Fig), and the protein was a next-neighbor of PrP also in this cell model (not shown) [15].

### PrP is critical for EMT-dependent polysialylation of NCAM1

**Fig 4:**
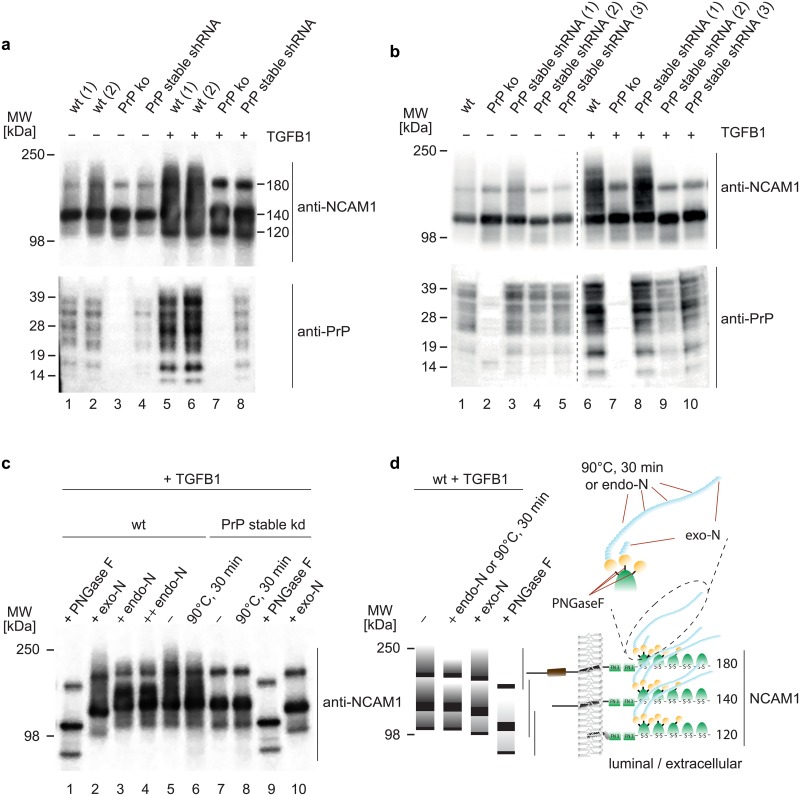
Stable PrP-deficiency prevents EMT-dependent polysialylation of NCAM1. (a) A post-translational modification of NCAM1 is missing in cells expressing no or low levels of PrP. Western blot analysis of selected NMuMG cell extracts revealed increased total levels of NCAM1 in all cell clones upon 48 h TGFB1 exposure. Whereas cells expressing wt levels of PrP give rise to a continuous pattern of NCAM1 signals, PrP-deficient cells exhibit more distinct NCAM1 bands, whose masses correspond to the expected masses of the three predominant NCAM1 isoforms. Note that the PrP^C^ band pattern observed in NMuMG cells tends to be more complex than the corresponding pattern in, for example, the Neuro2a cell model, possibly reflecting a greater heterogeneity of its N-glycans in these cells. (b) Screening of a larger number of stable PrP shRNA NMuMG clones further corroborated a direct correlation between PrP expression levels and post-translationally modified NCAM1 isoforms. Stable PrP shRNA clone 1, which exhibited no reduction in post-translationally-modified NCAM1 signals, turned out to express near wild-type levels of PrP, thereby establishing this clone as a false negative shRNA control. (c) Stable PrP-deficiency impairs polysialylation of NCAM1 at N-glycan acceptor sites. To characterize the post-translational NCAM1 modification lacking in PrP-deficient cells, extracts from wt or stable PrP kd NMuMG cells, which had been treated with TGFB1 for 48 h, were subjected to enzymatic digestion with glycosylases known to remove terminating sialic acids (exo-N), cut polysialic acid chains (endo-N) or hydrolyze the linkage of N-glycan groups to asparagine side-chains within ‘NxS/T’ acceptor sites (PNGase F). Note that complete removal of N-glycans abolishes the discriminating NCAM1 modification. (d) Interpretative panel of western blot bands observed in subpanel c. Red lines indicate expected cleavage sites for treatment conditions shown.

Validating the mass spectrometry results, TGFB1 treatment of wt NMuMG cells caused a pronounced upregulation of NCAM1 protein levels in western blot analyses. Intriguingly, high molecular mass (HMM) bands of diffuse appearance, which were reactive to the NCAM1 antibody and intensified during the course of EMT in wt NMuMG cells, were largely absent in PrP-deficient cells (Fig 4a). Ruling out clonal idiosyncracies as an explanation for their disappearance, the HMM bands were missing both in CRISPR-Cas9-generated PrP ko clones and in several stable PrP shRNA kd clones (Fig 4b).

NCAM1 is known to undergo several well-characterized post-translational modifications, and is the predominant acceptor of polysialic acid modifications in the brain. Treatment of cellular extracts with PNGaseF completely removed the HMM smearing, consistent with it originating from N-glycan moieties (Fig 4c). Treatment with endoneuraminidase (endo-N), a class of enzyme specific for the endoglycolysis of (2→8)-α-sialosyl linkages, reduced the most slowly migrating HMM signals only observed in PrP-expressing cells, indicating that polysialic acids are responsible for their presence. A less pronounced but visible reduction of the HMM smear was achieved by 90°C heat treatment, a method known to partially remove polysialic acid residues [31]. Finally, treatment with an exo-neuraminidase, which removes terminal sialic acid residues but does not remove longer polysialic acid chains, had only a minor effect on the smeared HMM component of NCAM1 signals but caused a slight shift of the most prominent NCAM1 bands to faster migrating species in both PrP-expressing and PrP-deficient cells (Fig 4c). These data established that PrP does not affect the addition of N-glycan core structures, including the addition of short terminal sialic acids. Instead, PrP’s influence on NCAM1 glycosylation relates specifically to its polysialylation (Fig 4d).

### PrP regulates NCAM1 polysialylation at the level of polyST transcription

**Fig 5:**
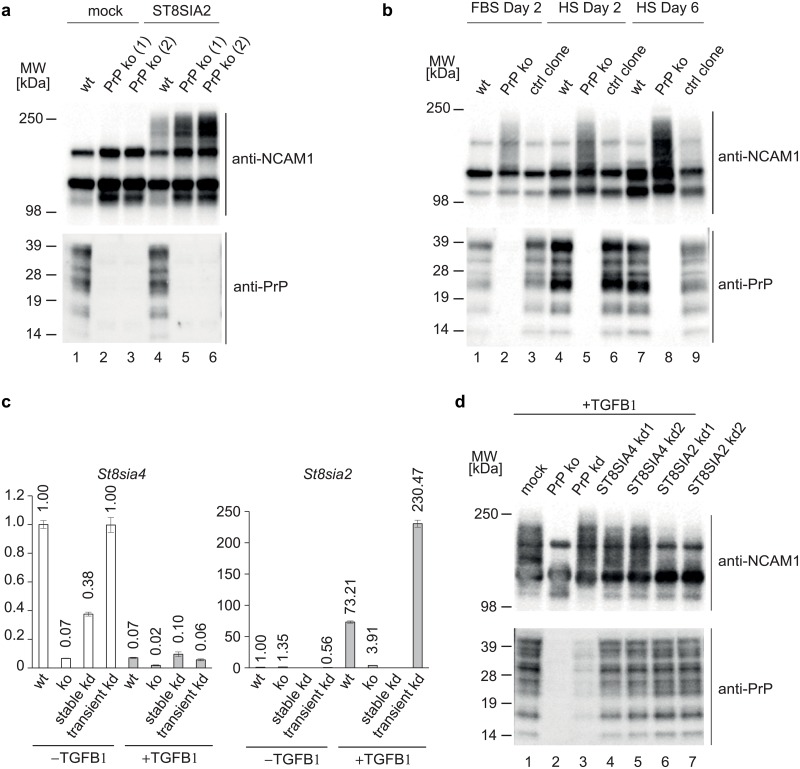
PrP deficiency prevents EMT-dependent NCAM1 polysialylation by inhibiting transcriptional activation of ST8SIA2 gene. (a) PrP is not required for NCAM1 polysialylation in N2a cells made to express ST8SIA2 from a heterologous expression plasmid. Note that higher levels of PSA-NCAM1 in CRISPR-Cas9-based PrP knockout N2a cells reflect higher levels of NCAM1 substrate in the PrP ko cell clones. (b) CRISPR-Cas9-based knockout of PrP in muscle C2C12 myocytes has little effect on total NCAM1 levels but causes profound upregulation of NCAM1 polysialylation before or throughout myotube differentiation. The control represents a C2C12 cell clone, which underwent all steps of CRISPR-Cas9 manipulation as the positive PrP ko clone but did not give rise to PrP ablation. FBS, cells grown in fetal bovine serum; HS, cells grown in horse serum (known to induce myotube formation). (c) Stable PrP ko or kd, but not transient PrP kd, impairs upregulation of ST8SIA2 transcripts in NMuMG cells in response to TGFB1 exposure. Note the different ordinate scales of subpanels. (d) ST8SIA2 is the polyST primarily responsible for EMT-dependent NCAM1 polysialylation in the NMuMG cell model. Transient kd of ST8SIA2 in NMuMG cells mimics stable PrP-deficiency with regard to its inhibition of NCAM1 polysialylation. Note that in contrast to the stable PrP knockout or kd, transient PrP-kd does not interfere with NCAM1 polysialylation.

Polysialic acids can be attached to N-glycans on NCAM1 by two polysialyltransferases (polyST), ST8SIA2 (STX) [32] and ST8SIA4 (PST) [33], which share 59% amino acid sequence identity [34]. To investigate if PrP directs polySTs to NCAM1 to facilitate its polysialylation, we made use of the N2a cell model, which was known to lack both polySTs [35]. Previously described PrP ko N2a clones [29] provided a useful paradigm for testing the possible contribution of PrP to NCAM1 polysialylation in a reconstitution experiment. As expected, western blot analyses confirmed the absence of PSA-NCAM1 in wt N2a cells, but also revealed that steady-state levels of NCAM1 expression were higher in PrP ko clones than in wt cells (Fig 5a, **lanes 1–3**). As planned, the transient heterologous expression of ST8SIA2 or ST8SIA4 (not shown) from expression plasmids rescued polysialylation of NCAM1 in these cells (Fig 5a, **lanes 4–6**). However, in contrast to expectations, NCAM1 polysialylation was not prevented in PrP ko cells but reached even higher levels—the latter presumably a consequence of the higher levels of NCAM1 substrate expressed in these clones.

To explore the scope of PrP’s influence on NCAM1 polysialylation, we next compared PSA-NCAM1 levels in selected brain regions and sciatic nerves in littermates of heterozygote PrP^0/wt^ intercrosses. This experiment revealed no apparent differences in the average steady-state PSA-NCAM1 levels in PrP-deficient tissues (**panel a in** S2 Fig), arguing that the effect of PrP on NCAM1 polysialylation might be unique to NMuMG cells, or could be restricted to cells undergoing specific cellular morphogenesis programs. Thus, before moving to further mechanistic investigations, analyses were extended to C2C12 cells, a muscle cell morphogenesis model, known to express both PSA-NCAM1 and PrP during myotube formation [36,37]. In support of the notion that PrP regulates NCAM1 polysialylation more broadly during morphogenesis, yet contrasting observations made in NMuMG cells, CRISPR-Cas9-generated PrP ko C2C12 myocytes and myotubes exhibited a profound increase in PSA-NCAM1 levels relative to wt C2C12 cells (Fig 5b).

We next considered if the role of PrP in NCAM1 polysialylation might be mediated through transcriptional regulation of polySTs in specific cellular contexts. To explore this concept, we turned toward the relative quantitation of transcripts coding for ST8SIA2 and ST8SIA4 in wt and PrP-deficient NMuMG cells. These RT-PCR analyses confirmed the successful >60% reduction of PrP transcript levels in all three PrP deficiency models tested (**panel b in** S2 Fig). Strikingly, whereas levels of ST8SIA4 transcripts were relatively low in NMuMG cells and were further reduced 48 h after EMT induction, ST8SIA2 transcript levels were observed to increase >50-fold upon EMT induction in wt cells (note the non-identical axis scales in subpanels of Fig 5c) but not in stable PrP-deficient cells. These data established that PrP exerted its effect on NCAM1 polysialylation through transcriptional regulation of the ST8SIA2 gene. Pointing toward an interesting exception, NMuMG cells in which PrP transcripts had only been transiently reduced (see also below) did not exhibit a reduction but a three-fold increase in ST8SIA2 transcript levels compared to wt levels (Fig 5c).

To validate these findings, siRNAs directed toward ST8SIA2 or ST8SIA4 were transfected into NMuMG cells induced to undergo EMT, and the consequences of this manipulation for NCAM1 polysialylation were monitored by western blot analysis. As expected, the kd of ST8SIA2 with siRNAs robustly interfered with the appearance of the characteristically smeared HMM PSA-NCAM1 bands (Fig 5d, **lanes 6, 7**). In contrast, and consistent with RT-PCR results, transient siRNA-mediated PrP kd did not inhibit NCAM1 polysialylation, despite robust reduction of PrP protein levels, consolidating the observation that only the stable kd or ko of PrP manifests in a reprogramming of NMuMG cells that precludes NCAM1 polysialylation (Fig 5d, **lanes 2,3**). Finally, RT-PCR analyses of RNA preparations from C2C12 cells established that PrP ko cells exhibit more than tenfold higher levels of ST8SIA2 than wt controls, explaining the profound increase in PSA levels we had observed by western blot analyses in PrP-deficient C2C12 cells (**panel c in** S2 Fig). Taken together, these experiments identified ST8SIA2 transcriptional regulation as the mechanism by which PrP influences PSA-NCAM levels in both NMuMG and C2C12 cells.

### β-catenin contributes to differential ST8SIA2 expression

**Fig 6:**
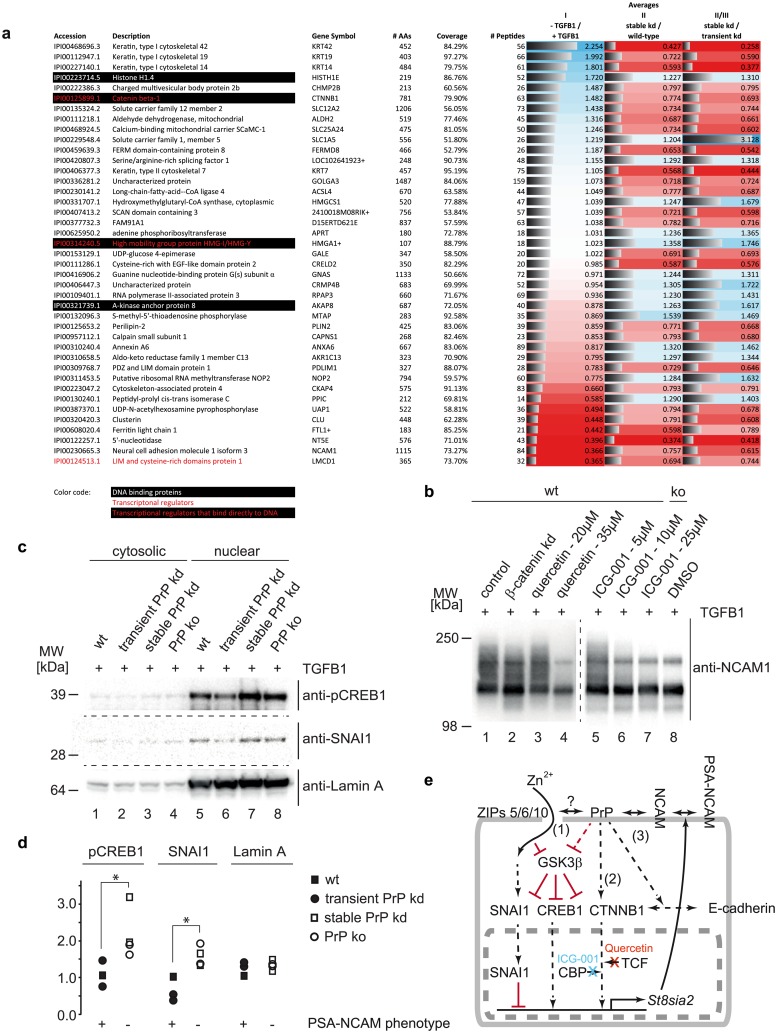
Inhibition of CTNNB1-dependent transcription phenocopies loss of PSA in NMuMG cells. (a) Comparison of global proteomes of stable PrP kd clones versus wt NMuMG cells and stable versus transient PrP-deficient cell clones. Of the total of 1421 proteins quantified in all global proteome analyses, relative levels of 41 proteins were changed by more than 20% in the direct comparisons. Four and three proteins had prior GO annotations, which identified them as ‘DNA binding’ and/or ‘Transcriptional regulators’. Based on these annotations, only β-catenin emerged as a DNA-binding transcriptional regulator whose levels are also changed during EMT. Note also that the level changes between stable kd cells and wt or transient kd NMuMG cells turned out to be equidirectional for all proteins whose levels changed more than 20%. (b) Transient kd of CTNNB1 or inhibitor-based disruption of protein-protein interactions between CTNNB1 and TCF or CBP reduces polysialylation of NCAM1. (c) Stable PrP ko or kd in NMuMG cells altered nuclear levels of SNAI1 and p133-CREB, developmental transcription factors known to interact with CTNNB1. Lamin A served as a nuclear reporter protein in these experiments, indicating both enrichment levels of nuclear fractions and equal protein loading. (d) Quantitation of nuclear levels of SNAI1 and p133-CREB in stable PrP ko or kd NMuMG clones versus wild-type or transient PrP kd NMuMG cells. The asterisks indicate significant differences in levels of SNAI1 (p = 0.029) and p133-CREB (p = 0.029) in cells that support or are impaired in NCAM1 polysialylation during EMT. (e) Cartoon depicting signaling pathways which may underlie differences in NCAM1 polysialylation in stable PrP-deficient cells.

We next set out to identify components of the PrP-dependent transcriptional machinery that govern ST8SIA2 gene regulation. The analysis capitalized on the observation that transient and stable PrP kd NMuMG cells differed with regard to NCAM1 polysialylation, and utilized this insight as a filter criterion for limiting the number of proteins-of-interest. Thus, based on the polysialylation phenotypes we had observed, suitable candidates would have to exhibit divergent expression levels in comparisons of stable PrP kd NMuMG clones (with impaired NCAM1 polysialylation) versus wt NMuMG cells or their transient PrP kd derivatives (with NCAM1 polysialylation intact). A combined analysis of datasets I to III revealed 41 proteins that fulfilled this criterion (deviating by more than 20% between TGFB1-treated stable PrP kd cells and TGFB1-treated wt cells or their transient PrP kd derivatives) and passed the previously described quality thresholds for confident quantifications (Fig 6a). Two of these proteins, namely high mobility group protein HMG-I/HMG-Y (HMGA1) and β-catenin (CTNNB1), had GO annotations that indicated them to be both DNA binding proteins and transcriptional regulators, but only CTNNB1 was also robustly induced during EMT (**panel b in** S1 Fig).

To determine if changes to CTNNB1 expression products are merely correlative or contribute to differences in NCAM1 polysialylation, TGFB1-treated wt NMuMG cells were subject to three alternative strategies known to reduce CTNNB1-dependent transcriptional activity, namely siRNA mediated kd of *Ctnnb1*-transcripts and pharmacological inhibition of CTNNB1 interactions with two of its known transcriptional partners. More specifically, the drugs quercetin [38] and ICG-001 [39] were used to interfere with interactions between CTNNB1 and transcription factor 4 (TCF4) or CREB binding protein (CBP), respectively. Consistent with an activating role of CTNNB1 in ST8SIA2 transcription, all three of these approaches reduced the characteristic smearing of PSA-NCAM1 signals, emphasizing to varying degrees the detection of non-polysialylated NCAM1 isoforms (Fig 6b). Finally, because CTNNB1 is known to operate in the context of multi-protein transcriptional assemblies, we also considered participation of other transcriptional regulators and extended the analysis to SNAI1 and CREB1, two developmental regulators of transcription, which are (i) known to co-operate with CTNNB1 in a subset of paradigms, and (ii) whose levels are, like the levels of CTNNB1 itself, controlled by GSK3β-dependent phosphorylation [40,41]. Indicative of a scenario, whereby these known CTNNB1-interactors may indeed contribute to *Sta8sia2* transcription in stable PrP-deficient cells, levels of SNAI1 and CREB1 (detected on the basis of a well-known S133 phospho-epitope), segregated with an inhibitory role in the polysialylation phenotype, i.e., were observed to be significantly higher in TGFB1-treated cells, which fail to induce ST8SIA2 transcription (Fig 6c and 6d).

## Discussion

This study investigated a possible role of PrP^C^ in EMT and uncovered that stable PrP-deficient cells undergo a proteome shift which affects preferentially the levels of proteins associated with this morphogenesis program, thereby precluding its proper execution and perturbing NCAM1 polysialylation. Instrumental for the success of this project was the application of an advanced proteomics discovery platform, which pointed toward NCAM1 and CTNNB1 as proteins whose levels were robustly altered during EMT and affected by stable PrP deficiency. Surprisingly, PrP’s influence on NCAM1 polysialylation was observed to not depend on PrP directing polySTs to its next neighbor NCAM1 but relies on signaling that involves the PrP-dependent transcriptional regulation of ST8SIA2. The striking differences in NCAM1 polysialylation observed in transient versus stable PrP-deficient cells brought to the fore the impact alternative kd methodologies can have on experiment outcome, a design element deserving attention also in other kd studies. Here, it helped us to identify CTNNB1 as a cellular factor that contributes to the PrP-dependent transcriptional regulation of ST8SIA2.

### The PrP-ST8SIA2-NCAM1 signaling loop

The NMuMG model emerged as a promising paradigm for studying signaling upstream and downstream of PrP in this work. For a protein whose expression has long been considered to be under the control of ‘housekeeping’ promoter elements [42,43,44], an unexpected observation was the more than tenfold transcriptional upregulation of endogenous PrP that accompanies EMT in NMuMG cells. Insights into the cellular programs that govern PrP expression are expected to provide novel angles for devising prion disease interventions [45]. Experimental models that facilitate the dissection of these upstream signaling pathways are therefore urgently needed. Although several transcription factors are known to act upstream of PrP in some experimental paradigms [46], the details of the regulation of the *Prnp* promoter and the broader context that drives its transcription have remained murky. Similarly, whereas the next-neighbor relationship of PrP and NCAM had been established for some time [15], the coinciding upregulation of their expression during EMT was not anticipated. Even more surprising is the existence of a signaling loop from PrP through ST8SIA2 to NCAM1. However, in a sense, this loop is no different from other signaling loops that exist in cells. For instance, ligand-receptor interactions, frequently, translate protein-protein interactions at the plasma membrane into transcriptional responses, which, in turn, provide post-translational feedback to one of the partners that triggered the loop (e.g., by ligand cleavage or receptor phosphorylation). Close investigation of molecular rearrangements at the plasma membrane will be required to determine if recognition of NCAM1 by PrP is required to trigger this feedback loop *in vivo*.

Interestingly, the consequence of PrP ko with regard to ST8SIA2 transcription followed opposite trends in NMuMG and C2C12 cells, suggesting that the signaling steps, which connect the two events, are either not conserved in the two cell models, or may integrate input from other factors. With CTNNB1 serving a dual role in the structural organization of adherens junctions and transcriptional gene regulation, its identification as a protein whose levels are reduced in stable PrP-deficient NMuMG cells was foreshadowed by the disrupted cadherin distribution we observed in the absence of PrP (Fig 1e). The subsequent transient kd or pharmacological inhibition of CTNNB1 established that this transcription factor contributes to the PrP-dependent transcriptional regulation of ST8SIA2. Along with the repressor SNAI1, CTNNB1 and CREB1 represent some of the best studied developmental transcriptional regulators. Indeed, several EMT-like phenotypes observed in ZIP-deficient paradigms have been phenocopied by SNAI1 kd [20,47]. The stability of all three factors is negatively controlled by GSK3β-mediated phosphorylation, which can direct them to the proteasome [48]. For the transcriptional regulation of specific target genes, these proteins associate with additional auxiliary factors, such as the transcriptional activator TCF4 and the co-activator CBP, in dynamic constellations that vary with time and cellular context. Importantly, the net effect on the transcription of a specific gene depends on the precise composition of the transcriptional complexes assembled on its promoter elements, and whether or not they are dominated by a repressor or activator (Fig 6e). Thus, the opposite outcome of stable PrP-deficiency with regard to NCAM1 polysialylation in NMuMG or C2C12 cells may be borne in a subtle difference in the transcriptional complex driving ST8SIA2 transcription in the two cell lines.

Follow-up work with emphasis on transcriptional regulation is needed to conclusively reveal which regulatory component is most directly impacted by PrP and mediates its effect on polyST transcription. In any case, once the polyST has been expressed, there seems to be no need for PrP to be present in order to direct this enzyme to NCAM1 and facilitate its polysialylation, because stable ablation of PrP in the Neuro2a cell model did not interfere with NCAM1 polysialylation when the production of polySTs was ectopically driven from heterologous plasmids.

### The search for the physiological function of PrP

It is safe to assume that many of the diverse functions attributed to PrP in the literature represent consequences that arise from more than one of its interactions and require specific cellular contexts. Viewed in this manner, PrP’s involvement in EMT may be considered one more phenotype the protein can be linked to. Yet, this phenotype has some objective characteristics, which make it uniquely stand out: (1) Its identification was precipitated by a research trajectory that built on insights gained from studying PrP’s relationship to its evolutionary ancestors. (2) Its key components, NCAM polysialylation and PrP, evolved around the same time and are restricted to vertebrates. (3) It reflects a role of PrP, which in NMuMG cells is preceded by a more than tenfold transcriptional upregulation of its protein levels, indicative that it might not represent a bystander phenomenon but is central to its biological role. (4) It centers on NCAM, the predominant next-neighbor of mature PrP at the plasma membrane. (5) Its consequences are profound, as it generates (at least in the two paradigms studied) a major change to a post-translational modification, which can by itself influence several aspects of cell biology, including cellular migration, cell-cell adhesion, and others (discussed further below).

It is to be anticipated that the influence of PrP on NCAM polysialylation is not restricted to the cell models or morphogenetic EMT program studied here. It is increasingly recognized that cells put to use branches of this program also in other cell fate decisions. We have, for instance, already shown that the effect of PrP on NCAM1 polysialylation is also observed in C2C12 myotube differentiation. Prominent cellular activities that have been associated with NCAM1 polysialylation are cell migration, neurite outgrowth [49,50], including mossy fiber pathfinding [51], hematopoietic stem cell differentiation [52,53], as well as AMPA [54] and NMDA receptor modulation [55,56]. NCAM1 polysialylation has further been shown to play a role in circadian rhythm regulation [57,58,59,60,61], myelin repair [62,63,64,65] and neurogenesis in both the subventricular zone and the dentate gyrus within the hippocampal formation [66,67,68]. Readers versed in the literature on PrP function will recognize that roles in all of these biological processes have also been attributed to PrP [2,69] (S5 Table). Careful further investigation will tell, which of the reported phenotypes will hold up upon close scrutiny, and if the connection between PrP (and possibly other ZIPs with a PrP-like ectodomain) and polysialylation is sufficient to explain them. Importantly, if it turns out that the ability of PrP to regulate polysialylation is central to its function, we submit that it still would not constitute a satisfying description of the function of PrP. In our view, a meaningful functional annotation of a protein encompasses both the larger program its activity/presence contributes to and the immediate molecular mechanism by which it contributes. On this account, regulation of polysialylation might turn out to be the larger program PrP serves in several paradigms but the detailed mechanism of its contribution remains to be elucidated.

## Conclusion

This study tied PrP to the field of protein polysialylation in two specific cellular morphogenesis programs, possibly foreshadowing a broader role of PrP as a regulator of polysialyation also in other cellular contexts. Detailed analyses will be needed to corroborate or refute the merits of this conceptual framework. Incidentally, the catalyzing initial discoveries in these two previously separate fields of investigation, namely the identification of the prion protein and the first description of NCAM polysialylation, were both made in 1982 [1,31]. Since then, both fields of study have matured side-by-side, with hundreds of articles published to date, and have given rise to a wealth of tools and models. We hope that the connection between PrP and NCAM1 polysialylation will stimulate progress in both areas of study.

## Materials and Methods

### Inhibitors, proteins, plasmids and antibodies

The chemicals quercetin (Q4951; Sigma-Aldrich, ON, Canada) and ICG-001 (S2662; Selleck chemical, TX, USA) were dissolved in DMSO and added to the cells 15h before co-treatment with TGFB1. Unless indicated otherwise, human recombinant TGFB1 (240-B; R&D Systems, MN, USA) was added to cells at a final concentration of 6.4 ng/ml for 48h.

The plasmids coding for ST8SIA2 (MR205823) and ST8SIA4 (MR205502) were purchased from Origene (MD, USA). Transient knockdowns were achieved with SilencerSelect siRNAs against *Prnp* (s72188; Life Technologies) and *Ctnnb1* (s63418; Life Technologies). ON-TARGETplus SMARTpools were obtained from GE HealthCare (ON, Canada) to target *St8sia2* (L-042781-01-0005) and *St8sia4* (L-044724-01-0005) transcripts.

Immunoblotting made use of antibodies against NCAM1 (1:6666, 556324 or 556325; BD Biosciences, ON, Canada), PrP (1:2000, A03213; Bertin Pharma, France), E-cadherin (1:4000, 3195; Cell signaling, MA, USA), p-CREB (1:1000, 9198; Cell Signaling), SNAI1 (1:1000, 3879; Cell Signaling) and Lamin A (1:1000, 26300; Abcam). For the microscopy analyses, the Alexa Fluor 488 goat anti-rabbit IgG (1:200, A31627) and Alexa Fluor 647 phalloidin (1:200, A22287) antibodies were purchased from Life Technologies (ON, Canada).

### Cell lines and culture conditions and transfections

C2C12 mouse myoblast (CRL-1772) and Neuro-2a mouse neuroblast (CCL-131) cells were purchased from American Type Culture Collection (ATCC, Rockville, MD). The mouse mammary gland epithelial cell line, NMuMG (CRL-1636), was a kind gift from Dr. Jeff Wrana (University of Toronto, Toronto, ON). The cells were maintained as per the ATCC’s recommendations. Briefly, (Dulbecco’s) Modified Eagles medium was supplemented with 10% FBS (12484028; Life Technologies), 1% GlutaMAX (35050061; Life Technologies) and 1% antibiotic-antimycotic solution (15240062; Life Technologies). For NMuMG cells, human insulin solution (I9278; Sigma-Aldrich) was also added at a concentration of 10 μg/mL. To transfect the cells with cDNA or siRNAs, Lipofectamine LTX or RNAiMAX (Life Technologies) were used, respectively, as per the manufacturer’s recommendations. In order to differentiate the C2C12 myoblasts into forming myotubes, the cells were grown to confluency. The medium was at that time replaced by DMEM supplemented with 2% horse serum, 1% GlutaMAX and 1% antibiotic-antimycotic solution and replenished daily for the course of the treatment.

### CRISPR-Cas9 mediated knockout clones and transient or stable knockdown of PrP

The CRISPR/Cas9-based PrP ko clones were generated in NMuMG, C2C12 and N2a cell models by non-homologous end-joining of a double-strand cut introduced into *Prnp* Exon 3. The two NMuMG PrP ko clones were characterized by a deletion of 2 and 4 (or 5 and 47) nucleotides within the two *Prnp* alleles present in this cell line, which caused reading frame-shifts and led to premature nonsense codons. All PrP ko clones had been characterized and described before [29]. Whereas the stable PrP kd clone emerged from a random genomic integration of a PrP-specific shRNA silencing plasmid that also encoded for a puromycin selection marker [29], the transient kd was achieved by transfection of PrP-specific siRNAs.

### Western blot analyses

Cells were lysed in 1% NP-40, 50 mM Tris (pH 8.0) and 150 mM NaCl, supplemented with 1x Complete Protease Inhibitor Cocktail (11836170001; Roche, ON, Canada) and phosSTOP (04906837001; Roche). When required, subcellular fractionation was performed using a designated kit from Cell signaling (9038S) according to the manufacturer’s recommendations. The protein content was subsequently, adjusted using the bicinchoninic acid (BCA) assay. Samples were then separated on 4–12% or 12% Bis-Tris (Life Technologies) or 7% Tris-glycine gels (cast in-house). The proteins were then transferred to a 0.45 micron polyvinylidene fluoride membrane, blocked in 10% skimmed milk and probed overnight at 4°C with the respective antibody diluted in 5% skimmed milk. On the next day, the blots were incubated with HRP-conjugated anti-mouse (1:5000, 170–6516; BioRad, ON, Canada) or anti-rabbit (1:5000, 170–6515; BioRad) secondary antibodies and the ECL reagent (RPN2106; GE Healthcare). The signal was then visualized using a LI-COR Odyssey Fc digital imaging system (LI-COR Biosciences, NE, USA).

### Enzymatic characterization of post-translational modifications of NCAM1

Cell lysates adjusted for total protein content, were incubated overnight with 4 μL of PNGase F (P0705; New England Biolabs, ON, Canada), 1 or 4 μl of endo-N (AbC0020; ABC Scientific, CA, USA) or 4 μl of α2–3,6,8 neuraminidase (P0720; New England Biolabs) in a total reaction volume of 10 μl at 37°C. In each case, the reaction proceeded in the presence of buffer solutions, which were provided by the respective manufacturers together with enzymes.

### RT-PCR analyses

RNA preparations were analyzed by a TaqMan gene expression assay targeting mouse *Prnp*, *St8sia2* and *St8sia4* transcripts. Total RNA was extracted using the RNeasy Mini Kit (74104; Qiagen, ON, Canada) and reverse transcribed to cDNA with oligo dT primers or random primers using the AffinityScript Multiple Temperature cDNA Synthesis Kit (200436; Agilent Technologies, ON, Canada). RNA integrity was checked on an Agilent 2100 Bioanalyzer (all samples were with RIN>7). Real-time PCR analyses were then undertaken with these RNA preparations using TaqMan Universal Master Mix II (4440038, Life Technologies) in triplicate to generate technical amplification replicates of *Hprt* (Mm00446968_m1), *Tfrc* (Mm00441941_m1), *Prnp* (Mm00448389_m1), *St8sia4* (Mm01292231_m1) and *St8sia2* Mm01311039_m1) (Life Technologies) mRNAs. Amplification products were analyzed on an ABI Prism 7500 system (Life Technologies). Relative quantifications were based on the qBASE PLUS software (Biogazelle NV, Belgium) using the ddCt method after normalization to *Hprt* and *Tfrc* mRNAs. The relative expressions of target transcripts were scaled to samples derived from vehicle-treated NMuMG or C2C12 cells. Near-identical results were obtained with oligo dT primers and random primers.

### Co-immunofluorescence analyses

Cells were plated on glass coverslips the day before their exposure to TGFB1. Following their treatment, cells were fixed in 3% formaldehyde for 10 minutes and permeabilized with 0.2% Triton X-100 for 5 minutes. The blocking of unspecific binding sites was achieved by exposure to 3% bovine serum albumin for 30 minutes, before overnight incubation with the primary antibody at 4°C. The coverslips were then incubated with the fluorescently-conjugated secondary antibodies for 30 minutes and mounted on microscope slides using ProLong Gold or Diamond antifade reagents with DAPI (P36935 or P36962; Life Technologies). For image acquisition, Zeiss LSM700 or LSM710 confocal microscopes were used at excitation wavelengths of 405nm, 488nm and 639nm for DAPI, E-cadherin and F-actin staining, respectively.

### Sample preparation for comparative global proteomics analysis

Sample preparation and steps for the global proteome analyses were identical to those described in a recent report [29]. The covalent labeling of tryptic peptides in three biological replicate samples and three controls with the 6-plex amine-reactive tandem mass tag (TMT) labeling kit reagents followed manufacturer instructions (Thermo Fisher Scientific, MA, USA).

### Global proteome analyses

The mass spectrometry component of the global proteome analyses followed closely a previously described methodology [29]. Briefly, TMT-labeled peptide mixtures within each of the three datasets (I to III) were combined and subjected to reversed phase separation on a 25 cm nanobore column (Acclaim PepMap RSLC C18, 2 μm, 100 Å, 75 μm i.d.) using a 4 hr linear gradient of 2–95% acetonitrile in 0.1% formic acid, controlled by a split-free nanoflow liquid chromatography system (Easy nLC 1100, Thermo Fisher Scientific), which was pumping at a flow rate of 300 nL/min. The column eluent was sent to a 10 μm emitter tip for micro-ionspray ionization, and positively charged peptides were analyzed by an Orbitrap Fusion Tribrid mass spectrometer using a data-dependent acquisition method. This method conducted (i) a high resolution orbitrap survey scan, (ii) fragmented the most intense parent ions by CID for the identification of peptides in the ion trap, and (iii) detected TMT signature ions from the 10 most intense peptide fragments upon their collision with high-energy nitrogen atoms (HCD).

### Post-acquisition and statistical analyses of global proteome datasets

Mass spectrometry raw data files were analyzed by Mascot (Version 2.4; Matrix Science Ltd, London, UK) and Sequest HT search engines embedded within Proteome Discoverer software (Version 1.4; Thermo Fisher Scientific). All PSMs were based on queries of the mouse international protein index (IPI) (release 3.87). Tolerance filters of 0.1 Da and 50 ppm were applied for searches of parent spectra and tandem MS spectra, respectively (note that scatter plots depicting precursor mass errors as a function of peptides scores indicated that observed masses of PSMs, which passed stringency criteria, were largely within 10 ppm of theoretical masses). Database searches were configured to allow for up to two missed tryptic cleavages. Because 4-vinylpyridine was used as the alkylating agent, the search was configured to assume all cysteine side-chains were pyridylethyl-derivatized. Variable modifications considered were TMT reagent modifications of primary amines, phosphorylations of serines, theonines and tyrosines, deamidations of glutamine and asparagine, and oxidations of methionines. For relative quantitation the low mass TMT signature ion distributions were interpreted by an algorithm embedded in the Proteome Discoverer software, which also generated raw data graphs depicting TMT ratios (modified to produce S1 Fig). Statistical analyses of global proteomes were conducted with PEAKS (Version 6.0; Bioinformatics Solutions Inc., Waterloo, Ontario, Canada) and ProteinCenter (Thermo Fisher Scientific) software packages. A stringent false discovery rate (FDR) of 0.5% was set as the initial filter, which had to be passed by all PSMs. Because a reliable relative protein quantitation was critical for the interpretation of data, a subsequent filtering process eliminated all proteins, which were not identified and quantified on the basis of at least three PSMs for which TMT signature ion distributions were available. The application of this filter eliminated false-positive identifications by exceeding widely applied thresholds for protein group inclusion, which typically require assignments of two PSMs per protein group for confident identifications. KEGG pathway and GO ‘biological process’ annotations were determined by a ‘Statistical analysis’ algorithm within ProteinCenter (Thermo Fisher Scientific), which computed the respective significance scores by comparing annotations of proteins of interest against the combined list of 6124 proteins detected in global proteome datasets I to III. Because three biological replicates of TGFB1-treated wt NMuMG cells were common to all three proteomics analyses conducted, their global proteomes served as a reference against which all other datasets were compared. The Kruskal-Wallis test was applied to identify whether there is significant differences in the TMT-based relative ratios of proteins amongst the two sets of three biological replicates within each dataset (S1 Table). Since we could not assume Gaussian distributions for TMT ratios, this analysis was based on a non-parametric version of the test (IBM SPSS Statistics, version 20, NY, USA). A Spearman correlation analysis was conducted to determine whether there is a significant correlation between the TMT levels of the 57 proteins whose relative expression levels were most altered during EMT (dataset I) and following stable kd of PrP (dataset II) (Fig 3 and S2 Table). The mass spectrometry proteomics data have been deposited to the ProteomeXchange Consortium [70] via the PRIDE partner repository [71] with the dataset identifier PXD001875.

## Supporting Information

S1 Fig TMT enrichment plots depicting representative protein quantitations from datasets I and II. The plots exemplify TMT signature ion ratios for proteins whose expression levels (a) increased (NCAM1), (b) decreased (catenin beta 1), or (c) were unchanged (tubulin alpha 1) during 48 h exposure of cells to TGFB1 (dataset I). Each red or blue circle represents the TMT-based relative abundance ratio determined for one peptide. Levels of both NCAM1 and catenin beta 1 but not tubulin alpha 1 were lower in TGFB1-treated stable PrP kd cells than in wt NMuMG cells (dataset II). Note the high reproducibility between biological replicates. See legend at the bottom of the page for symbol explanations.
(EPS)

S2 Fig PrP knockout does not impair overall brain levels of PSA-NCAM1 but affects levels of *St8Sia2* transcripts in C2C12 myotube morphogenesis model. (a) Levels of PSA-NCAM1 are not changed in selected brain regions or sciatic nerves of 3 to 8 months old PrP knockout mice. (b) Levels of *Prnp* transcript levels following stable versus transient transfection of PrP-specific shRNAs or siRNAs, respectively. Note that the shRNA-based knockdown did not affect the very low base levels of PrP transcripts in untreated NMuMG cells (see also Fig 4b, **lanes 1, 4 and 5**) but its effect comes to the fore in TGFB1-treated cells (see also Fig 4b, **lanes 6, 9 and 10**). (c) Stable PrP ko in C2C12 cells causes upregulation of ST8SIA2 transcripts. FBS, fetal bovine serum; HS, horse serum.
(EPS)

S1 Table Proteins detected and quantified in datasets I to III on the basis of at least three TMT signature ion profiles (entries are sorted by their level of enrichment during EMT). (PDF)

S2 Table Top 200 proteins exhibiting most pronounced differences in expression before and after 48 hours TGFB1 treatment in wt NMuMG cells (extracted from dataset I). (PDF)

S3 Table Top 200 proteins exhibiting most pronounced differences in expression levels in direct comparison of 48 hours TGFB1-treated stable PrP kd versus wt cells (extracted from dataset II). (PDF)

S4 Table Overlap of top 200 proteins undergoing the most pronounced level changes during 48 hour TGFB1 treatment (dataset I) AND top 200 proteins observed at most divergent levels in a direct comparison of 48 hour TGFB1-treated stable PrP kd and wt cells (dataset II). (PDF)

S5 Table Similarities amongst prior independent observations of PrP- and PSA-NCAM-related phenotypes. (PDF)
