# Supplementary material for: The Prion Protein Controls Polysialylation of Neural Cell Adhesion Molecule 1 during Cellular Morphogenesis
Source: PLoS One. 2015 Aug 19;10(8):e0133741. doi: 10.1371/journal.pone.0133741 (PMC4546001; doi:10.1371/journal.pone.0133741)
Supplement: S1 Table — (PDF) [file pone.0133741.s003.pdf]

S1 Table: Proteins detected and quantified in datasets I to III on the basis of at least three TMT signature ion profiles (entries are sorted by their level of enrichment during EMT)

Accession	Original database description	Modified description	Coverage	# Proteins	# Unique Peptides	# Peptides	# PSMs	[illegible]
IPI00131547.1	Plasminogen activator inhibitor 1	Plasminogen activator inhibitor 1	82.84%	1	10	35	262	[illegible]
IPI00856302.1	Isoform 6 of Palladin	Palladin	61.90%	7	9	91	515	[illegible]
IPI00134746.5	Argininosuccinate synthase	Argininosuccinate synthase	77.67%	1	17	45	458	[illegible]
IPI00124513.1	LIM and cysteine-rich domains protein 1	LIM and cysteine-rich domains protein 1	73.70%	1	5	32	158	[illegible]
IPI00230665.3	neural cell adhesion molecule 1 isoform 3	Neural cell adhesion molecule 1 isoform 3	73.27%	4	14	84	731	[illegible]
IPI00331612.3	High mobility group protein HMGI-C	High mobility group protein HMGI-C	75.93%	2	5	11	452	[illegible]
IPI00122257.1	5'-nucleotidase	5'-nucleotidase	71.01%	2	7	43	175	[illegible]
IPI00265705.2	Isoform 2 of Mitogen-activated protein kinase kinase kinase MLT	Mitogen-activated protein kinase kinase kinase MLT	36.56%	1	2	19	104	[illegible]
IPI00128209.3	Isoform 1 of Adenylate kinase isoenzyme 1	Adenylate kinase isoenzyme 1	91.75%	3	6	22	124	[illegible]
IPI00608020.4	ferritin light chain 1	Ferritin light chain 1	85.25%	3	5	21	301	[illegible]
IPI00320420.3	Clusterin	Clusterin	62.28%	1	10	39	249	[illegible]
IPI00281011.7	MARCKS-related protein	MARCKS-related protein	91.50%	1	8	13	297	[illegible]
IPI00670967.3	Fermitin family homolog 1	Fermitin family homolog 1	70.90%	2	4	47	171	[illegible]
IPI00277930.7	Putative uncharacterized protein	Putative uncharacterized protein	60.17%	3	10	28	374	[illegible]
IPI00970075.1	Uncharacterized protein	Uncharacterized protein	81.40%	3	11	33	311	[illegible]
IPI00387370.1	Isoform AGX2 of UDP-N-acetylhexosamine pyrophosphorylase	UDP-N-acetylhexosamine pyrophosphorylase	58.81%	5	9	36	214	[illegible]
IPI00275753.1	Scr3	Scr3	56.74%	3	7	33	408	[illegible]
IPI00123758.3	procollagen-lysine,2-oxoglutarate 5-dioxygenase 2 isoform 1	Procollagen-lysine,2-oxoglutarate 5-dioxygenase 2 isoform 1	59.10%	2	15	61	364	[illegible]
IPI00114342.1	Hexokinase-2	Hexokinase-2	85.06%	2	15	88	388	[illegible]
IPI00120245.2	Integrin alpha-V	Integrin alpha-V	70.21%	2	14	85	536	[illegible]
IPI00123540.1	Tetraspanin-7	Tetraspanin-7	42.57%	2	1	10	158	[illegible]
IPI00130240.1	Peptidyl-prolyl cis-trans isomerase C	Peptidyl-prolyl cis-trans isomerase C	69.81%	1	3	14	109	[illegible]
IPI00399959.1	Isoform 2 of Prolyl 4-hydroxylase subunit alpha-1	Prolyl 4-hydroxylase subunit alpha-1	72.85%	3	11	39	311	[illegible]
IPI00756703.1	Isoform 2 of Serine/threonine-protein phosphatase 2B catalytic subunit alpha isoform	Serine/threonine-protein phosphatase 2B catalytic subunit alpha isoform	74.95%	12	6	40	185	[illegible]
IPI00830803.1	fibulin-2 isoform b	Fibulin-2 isoform b	56.05%	1	9	49	284	[illegible]
IPI00331598.2	Uncharacterized protein	Uncharacterized protein	60.58%	3	3	31	112	[illegible]
IPI00308785.1	Prostaglandin G/H synthase 2	Prostaglandin G/H synthase 2	61.09%	2	6	36	157	[illegible]
IPI00268377.7	Beta-parvin	Beta-parvin	58.36%	1	4	29	170	[illegible]
IPI00555103.2	protein MICAL-2 isoform A	MICAL-2 isoform A	68.78%	3	4	85	270	[illegible]
IPI00132474.3	Integrin beta-1	Integrin beta-1	75.56%	1	11	74	457	[illegible]
IPI00330289.4	Uncharacterized protein	Uncharacterized protein	56.97%	4	1	63	434	[illegible]
IPI00990293.1	band 4.1-like protein 2	Band 4.1-like protein 2	64.78%	5	3	83	628	[illegible]
IPI00124803.1	Zinc finger protein ZPR1	Zinc finger protein ZPR1	95.64%	3	14	46	252	[illegible]
IPI00118413.2	Uncharacterized protein	Uncharacterized protein	67.55%	1	8	90	329	[illegible]
IPI00123202.1	Ataxin-10	Ataxin-10	84.42%	1	12	47	401	[illegible]
IPI00320480.3	Sphingosine-1-phosphate lyase 1	Sphingosine-1-phosphate lyase 1	56.69%	2	6	44	179	[illegible]
IPI00133608.3	Isoform 1 of OCIA domain-containing protein 1	OCIA domain-containing protein 1	53.44%	3	1	15	55	[illegible]
IPI00223047.2	Cytoskeleton-associated protein 4	Cytoskeleton-associated protein 4	91.13%	6	32	83	753	[illegible]
IPI00457611.2	Isoform 1 of Septin-9	Septin-9	87.31%	3	24	72	845	[illegible]
IPI00114733.2	Serpin H1	Serpin H1	85.13%	1	21	53	511	[illegible]
IPI00654388.2	Isoform 1 of Leucine-rich repeat flightless-interacting protein 1	Leucine-rich repeat flightless-interacting protein 1	87.38%	5	16	72	938	[illegible]
IPI00395177.1	active breakpoint cluster region-related protein isoform 2	active breakpoint cluster region-related protein isoform 2	71.59%	6	6	72	314	[illegible]
IPI00126892.12	Putative uncharacterized protein	Putative uncharacterized protein	63.25%	1	2	18	65	[illegible]
[illegible]	[illegible]	[illegible]	[illegible]	[illegible]	[illegible]	[illegible]	[illegible]	[illegible]

IPI00313222.5	60S ribosomal protein L6	60S ribosomal protein L6	80.41%	4	21	47	569	0.936	42	24.1	1.039
IPI00115097.1	Coatomer subunit beta'	Coatomer subunit beta'	75.25%	1	22	79	478	0.881	24	22.2	1.009
IPI00265406.3	Phosphoribosylformylglycinamidine synthase	Phosphoribosylformylglycinamidine synthase	60.28%	1	8	70	246	0.896	5	12.3	0.970
IPI00108388.2	Probable ubiquitin carboxyl-terminal hydrolase FAF-X	Probable ubiquitin carboxyl-terminal hydrolase FAF-X	69.32%	8	16	176	659	0.862	7	20.5	1.037
IPI00131407.1	Proteasome subunit alpha type-5	Proteasome subunit alpha type-5	65.56%	1	10	24	184	0.934	8	20.0	0.918
IPI00330804.4	Heat shock protein HSP 90-alpha	Heat shock protein HSP 90-alpha	89.09%	3	30	88	1531	0.911	137	20.7	0.991
IPI00622235.5	Transitional endoplasmic reticulum ATPase	Transitional endoplasmic reticulum ATPase	84.86%	8	40	89	1662	0.922	85	26.4	0.980
IPI00282957.4	Isoform 1 of MAP7 domain-containing protein 1	MAP7 domain-containing protein 1	75.53%	4	13	99	518	0.925	9	5.8	1.037
IPI00415385.1	Isoform 1 of Bcl-2-associated transcription factor 1	Bcl-2-associated transcription factor 1	73.88%	5	17	110	484	0.934	25	15.2	1.035
IPI00111265.3	F-actin-capping protein subunit alpha-2	F-actin-capping protein subunit alpha-2	82.52%	1	6	24	268	1.013	12	20.8	0.905
IPI00116991.2	Histidyl-tRNA synthetase, cytoplasmic	Histidyl-tRNA synthetase, cytoplasmic	72.10%	1	12	63	293	0.955	10	18.3	0.963
IPI00132456.1	UPF0568 protein C14orf166 homolog	UPF0568 protein C14orf166 homolog	92.62%	3	12	31	287	0.946	16	12.6	1.013
IPI00153986.3	General transcription factor IIF subunit 1	General transcription factor IIF subunit 1	54.13%	1	9	39	179	0.954	4	13.9	0.850
IPI01026712.1	30 kDa protein	30 kDa protein	70.67%	2	13	25	572	0.920	32	26.4	0.949
IPI00130883.1	Putative RNA-binding protein 3	Putative RNA-binding protein 3	77.78%	2	3	18	201	0.967	12	26.8	0.939
IPI00114368.3	Vesicle-trafficking protein SEC22b	Vesicle-trafficking protein SEC22b	64.19%	2	6	22	256	0.951	16	20.0	1.057
IPI00749596.1	double-stranded RNA-binding protein Staufen homolog 1 isoform 1	Double-stranded RNA-binding protein Staufen homolog 1 isoform 1	83.84%	5	8	56	264	0.895	5	39.2	0.893
IPI00137331.7	Adenylyl cyclase-associated protein 1	Adenylyl cyclase-associated protein 1	75.32%	2	21	48	785	0.945	43	13.2	1.025
IPI00229845.3	Protein arginine N-methyltransferase 5	Protein arginine N-methyltransferase 5	55.89%	1	9	46	222	0.995	5	18.9	0.960
IPI00323660.1	Actin-like protein 6A	Actin-like protein 6A	69.23%	3	6	32	123	0.932	7	45.9	1.198
IPI00475248.3	Argonaute 2 protein (Fragment)	Argonaute 2 protein (Fragment)	59.60%	2	2	55	173	0.940	3	14.5	0.884
IPI00755843.1	Uncharacterized protein	Uncharacterized protein	62.79%	6	15	25	459	0.931	33	19.9	1.011
IPI00331174.5	T-complex protein 1 subunit eta	T-complex protein 1 subunit eta	97.79%	1	28	84	805	0.946	54	13.6	1.011
IPI00323881.2	Importin subunit beta-1	Importin subunit beta-1	63.47%	1	28	55	782	0.972	47	19.5	0.986
IPI00109401.1	RNA polymerase II-associated protein 3	RNA polymerase II-associated protein 3	71.67%	1	11	69	314	1.002	8	16.0	1.009
IPI00132475.2	Putative uncharacterized protein	Putative uncharacterized protein	69.10%	1	10	42	327	0.888	14	33.0	1.009
IPI00624746.3	inosine-5'-monophosphate dehydrogenase 2-like	Inosine-5'-monophosphate dehydrogenase 2-like	69.30%	2	19	50	366	0.909	26	21.8	0.992
IPI00116877.5	transportin-2	Transportin-2	54.29%	2	3	48	223	0.926	8	9.2	1.027
IPI00116283.1	T-complex protein 1 subunit gamma	T-complex protein 1 subunit gamma	97.43%	1	27	80	869	0.933	53	17.2	1.036
IPI00133700.2	Vacuolar protein sorting-associated protein 4B	Vacuolar protein sorting-associated protein 4B	66.44%	2	7	36	231	0.959	13	31.6	0.887
IPI00108791.2	FAS-associated factor 1	FAS-associated factor 1	72.57%	1	7	57	278	0.932	6	26.3	0.865
IPI00173343.3	Oligoribonuclease, mitochondrial (Fragment)	Oligoribonuclease, mitochondrial (Fragment)	66.67%	1	2	20	89	0.892	4	14.5	0.933
IPI01023315.1	Uncharacterized protein (Fragment)	Uncharacterized protein (Fragment)	78.70%	3	18	94	799	0.949	42	26.1	1.016
IPI00165694.1	Isoform 1 of Mitochondrial import receptor subunit TOM34	Mitochondrial import receptor subunit TOM34	81.55%	2	4	37	102	0.874	3	27.0	0.964
IPI00223055.3	glutaminyl-tRNA synthetase isoform 1	Glutaminyl-tRNA synthetase isoform 1	74.84%	3	15	67	198	0.931	3	12.2	1.051
IPI00124700.1	Transferrin receptor protein 1	Transferrin receptor protein 1	74.31%	2	15	72	521	0.890	37	25.5	0.949
IPI00990800.1	Uncharacterized protein	Uncharacterized protein	73.90%	3	5	90	364	0.888	3	4.9	0.936
IPI00928541.1	Isoform 2 of RNA-binding protein 25	RNA-binding protein 25	41.53%	3	11	50	274	0.896	10	31.2	1.018
IPI00313558.5	Coatomer subunit delta	Coatomer subunit delta	78.67%	1	14	62	400	0.928	25	18.9	0.987
IPI00309035.2	Dolichyl-diphosphooligosaccharide--protein glycosyltransferase subunit 1	Dolichyl-diphosphooligosaccharide--protein glycosyltransferase subunit 1	67.60%	1	22	59	451	0.915	24	10.7	1.033

IPI00114375.2	Dihydropyrimidinase-related protein 2	Dihydropyrimidinase-related protein 2	81.29%	2	24	52	1055
IPI00120691.4	Nucleolar RNA helicase 2	Nucleolar RNA helicase 2	85.19%	1	39	102	1108
IPI00625950.2	adenine phosphoribosyltransferase	adenine phosphoribosyltransferase	72.78%	2	11	18	84
IPI00307837.6	Elongation factor 1-alpha 1	Elongation factor 1-alpha 1	75.11%	5	15	53	1263
IPI00119618.1	Calnexin	Calnexin	58.54%	1	25	42	730
IPI00119458.7	Fructose-bisphosphate aldolase C	Fructose-bisphosphate aldolase C	93.66%	2	4	40	366
IPI00130409.1	Isoform 1 of Pre-mRNA-processing factor 6	Pre-mRNA-processing factor 6	71.94%	2	16	100	505
IPI00132958.1	Acyl-coenzyme A thioesterase 13	Acyl-coenzyme A thioesterase 13	67.14%	1	5	10	30
IPI00453599.2	Craniofacial development protein 1	Craniofacial development protein 1	50.85%	1	4	24	128
IPI00112004.1	Uncharacterized protein	Uncharacterized protein	76.42%	1	4	18	125
IPI00352475.3	28 kDa heat- and acid-stable phosphoprotein	28 kDa heat- and acid-stable phosphoprotein	90.06%	1	17	40	833
IPI00830797.1	splicing factor 1 isoform 2	Splicing factor 1 isoform 2	52.37%	6	10	47	419
IPI00849793.1	60S ribosomal protein L12	60S ribosomal protein L12	91.52%	2	7	24	305
IPI00857771.1	Uncharacterized protein	Uncharacterized protein	65.61%	8	18	151	724
IPI00132728.2	Isoform 1 of Cytochrome c1, heme protein, mitochondrial	Cytochrome c1, heme protein, mitochondrial	77.23%	2	3	24	142
IPI00420136.2	sorting nexin-3	Sorting nexin-3	88.89%	4	5	20	193
IPI00121322.2	Electron transfer flavoprotein-ubiquinone oxidoreductase, mitochondrial	Electron transfer flavoprotein-ubiquinone oxidoreductase, mitochondrial	56.01%	2	5	38	173
IPI00123870.2	Structural maintenance of chromosomes protein 1A	Structural maintenance of chromosomes protein 1A	79.64%	1	27	165	636
IPI00124223.3	Proteasome activator complex subunit 1	Proteasome activator complex subunit 1	86.35%	3	6	32	123
IPI00132473.3	Cleavage and polyadenylation specificity factor subunit 5	Cleavage and polyadenylation specificity factor subunit 5	75.77%	1	8	20	116
IPI00108125.4	Eukaryotic translation initiation factor 5A-1	Eukaryotic translation initiation factor 5A-1	94.16%	3	6	18	321
IPI00762269.1	Sorting nexin-12	Sorting nexin-12	76.36%	4	4	16	112
IPI00169463.1	Tubulin beta-2C chain	Tubulin beta-2C chain	76.63%	1	1	36	1049
IPI00224644.1	Isoform C of Serrate RNA effector molecule homolog	Serrate RNA effector molecule homolog	58.45%	4	15	63	496
IPI00120344.2	FACT complex subunit SPT16	FACT complex subunit SPT16	67.81%	2	20	101	627
IPI00308882.5	NADH-ubiquinone oxidoreductase 75 kDa subunit, mitochondrial	NADH-ubiquinone oxidoreductase 75 kDa subunit, mitochondrial	82.81%	1	16	73	335
IPI00229534.5	Myristoylated alanine-rich C-kinase substrate	Myristoylated alanine-rich C-kinase substrate	96.76%	1	13	22	1322
IPI00269481.7	Isoform 2 of F-actin-capping protein subunit beta	F-actin-capping protein subunit beta	86.76%	4	18	35	274
IPI00622811.2	Uncharacterized protein	Uncharacterized protein	87.99%	2	13	61	401
IPI00111013.1	Cathepsin D	Cathepsin D	58.29%	4	10	29	312
IPI00224575.1	Isoform 2 of Heterogeneous nuclear ribonucleoprotein K	Heterogeneous nuclear ribonucleoprotein K	89.87%	5	21	47	711
IPI00377732.3	Protein FAM91A1	FAM91A1	57.59%	1	3	63	269
IPI00753793.2	Isoform 2 of Spectrin alpha chain, brain	Spectrin alpha chain, brain	78.22%	3	3	226	1464
IPI00125460.1	ATP synthase-coupling factor 6, mitochondrial	ATP synthase-coupling factor 6, mitochondrial	98.15%	2	6	18	222
IPI00131224.1	Transcription elongation factor B polypeptide 2	Transcription elongation factor B polypeptide 2	98.31%	1	5	14	291

[illegible]

IPI01016218.1	serine/arginine repetitive matrix protein 1 isoform 1	Serine/arginine repetitive matrix protein 1 isoform 1	78.55%	7	13	144	644
IPI00554989.3	Peptidyl-prolyl cis-trans isomerase	Peptidyl-prolyl cis-trans isomerase	92.81%	1	4	29	438
IPI00113134.1	Superkiller viralicidic activity 2-like 2	Superkiller viralicidic activity 2-like 2	62.21%	2	12	86	375
IPI00110721.5	Isoform 1 of Glyoxalase domain-containing protein 4	Glyoxalase domain-containing protein 4	81.21%	5	11	29	162
IPI00321734.7	Lactoylglutathione lyase	Lactoylglutathione lyase	59.24%	1	5	14	90
IPI00224784.4	Prothymosin alpha	Prothymosin alpha	43.24%	1	6	9	344
IPI00755183.1	Uncharacterized protein	Uncharacterized protein	91.27%	6	7	25	184
IPI00380309.3	Isoform 1 of Putative RNA-binding protein Luc7-like 2	Putative RNA-binding protein Luc7-like 2	74.49%	8	5	51	296
IPI00990544.1	Glyceraldehyde-3-phosphate dehydrogenase	Glyceraldehyde-3-phosphate dehydrogenase	88.96%	3	1	39	799
IPI00225961.5	D-3-phosphoglycerate dehydrogenase	D-3-phosphoglycerate dehydrogenase	59.66%	2	12	43	465
IPI00137439.1	Dual specificity mitogen-activated protein kinase kinase 6	Dual specificity mitogen-activated protein kinase kinase 6	75.75%	2	1	32	132
IPI01027807.1	Uncharacterized protein	Uncharacterized protein	70.20%	2	21	79	584
IPI00124692.1	Transaldolase	Transaldolase	77.15%	1	15	39	351
IPI00122392.1	UPF0160 protein MYG1, mitochondrial	UPF0160 protein MYG1, mitochondrial	83.16%	1	5	33	214
IPI00882156.1	Uncharacterized protein	Uncharacterized protein	86.67%	2	6	50	258
IPI00407130.4	Isoform M2 of Pyruvate kinase isozymes M1/M2	Pyruvate kinase isozymes M1/M2	90.02%	2	12	80	1373
IPI00471441.1	Ptms protein	Ptms protein	66.35%	3	6	20	371
IPI00113362.2	Crk-like protein	Crk-like protein	64.03%	1	10	28	159
IPI00229548.4	Solute carrier family 1 (Neutral amino acid transporter), member 5	Solute carrier family 1 (Neutral amino acid transporter), member 5	51.80%	3	7	26	224
IPI00119714.2	Upstream binding transcription factor, RNA polymerase I, isoform CRA_b	Upstream binding transcription factor, RNA polymerase I, isoform CRA_b	67.82%	7	5	90	312
IPI00322209.5	Keratin, type II cytoskeletal 8	Keratin, type II cytoskeletal 8	97.76%	11	42	90	3235
IPI00115257.1	Isoform 1 of PC4 and SFRS1-interacting protein	PC4 and SFRS1-interacting protein	79.55%	3	10	64	280
IPI00453499.3	Isoleucyl-tRNA synthetase, mitochondrial	Isoleucyl-tRNA synthetase, mitochondrial	54.84%	3	6	68	224
IPI00273096.7	Aldose reductase-related protein 2	Aldose reductase-related protein 2	69.30%	4	4	28	136
IPI00318778.2	Calcium-binding protein 39	Calcium-binding protein 39	74.78%	1	10	38	194
IPI00125880.1	Protein kinase C and casein kinase substrate in neurons protein 2	Protein kinase C and casein kinase substrate in neurons protein 2	62.96%	1	11	42	204
IPI00129430.1	Splicing factor, proline- and glutamine-rich	Splicing factor, proline- and glutamine-rich	65.81%	1	21	71	641
IPI00131177.1	LETM1 and EF-hand domain-containing protein 1, mitochondrial	LETM1 and EF-hand domain-containing protein 1, mitochondrial	72.76%	1	7	77	279
IPI00387212.1	N-alpha-acetyltransferase 15, NatA auxiliary subunit	N-alpha-acetyltransferase 15, NatA auxiliary subunit	76.42%	1	14	96	379
IPI00124372.3	4-trimethylaminobutyraldehyde dehydrogenase	4-trimethylaminobutyraldehyde dehydrogenase	66.22%	1	4	31	94
IPI00123176.1	Glyceraldehyde-3-phosphate dehydrogenase	Glyceraldehyde-3-phosphate dehydrogenase	88.20%	3	3	38	463
IPI00112719.1	Delta-aminolevulinic acid dehydratase	Delta-aminolevulinic acid dehydratase	72.73%	1	9	19	178
IPI00338536.1	Succinate dehydrogenase [ubiquinone] iron-sulfur subunit, mitochondrial	Succinate dehydrogenase [ubiquinone] iron-sulfur subunit, mitochondrial	82.27%	1	8	43	203
IPI00407692.3	Isoform 1 of V-type proton ATPase catalytic subunit A	V-type proton ATPase catalytic subunit A	76.01%	1	20	45	348
IPI00230139.5	Peptidyl-prolyl cis-trans isomerase FKBP4	Peptidyl-prolyl cis-trans isomerase FKBP4	61.35%	2	17	44	547
IPI00881406.1	rho guanine nucleotide exchange factor 1 isoform b	Rho guanine nucleotide exchange factor 1 isoform b	65.06%	5	5	79	276
IPI00119114.2	Long-chain specific acyl-CoA dehydrogenase, mitochondrial	Long-chain specific acyl-CoA dehydrogenase, mitochondrial	72.33%	2	14	46	296
IPI00126338.6	Isoform Alpha of Lamina-associated polypeptide 2, isoforms alpha/zeta	Lamina-associated polypeptide 2, isoforms alpha/zeta	82.11%	1	7	76	542
IPI00338964.3	Isoform SERCA2B of Sarcoplasmic/endoplasmic reticulum calcium ATPase 2	Sarcoplasmic/endoplasmic reticulum calcium ATPase 2	57.47%	3	19	90	719
IPI00311654.4	Sarcoplasmic/endoplasmic reticulum calcium ATPase 1	Sarcoplasmic/endoplasmic reticulum calcium ATPase 1	56.44%	2	2	77	358
IPI00403336.4	Isoform 2 of Dynamin-like 120 kDa protein, mitochondrial	Dynamin-like 120 kDa protein, mitochondrial	70.11%	5	7	102	342
IPI00221402.7	Fructose-bisphosphate aldolase A	Fructose-bisphosphate aldolase A	79.12%	2	2	33	804
IPI00311461.1	V-type proton ATPase subunit H	V-type proton ATPase subunit H	86.54%	1	10	55	367
IPI00111258.4	major vault protein	Major vault protein	78.28%	3	33	90	769
IPI00266899.6	Peptidyl-prolyl cis-trans isomerase FKBP1A	Peptidyl-prolyl cis-trans isomerase FKBP1A	61.11%	2	2	13	64
IPI00468924.5	Calcium-binding mitochondrial carrier protein SCaMC-1	Calcium-binding mitochondrial carrier protein SCaMC-1	81.05%	1	13	50	306
IPI00263874.3	Phenylalanyl-tRNA synthetase alpha chain	Phenylalanyl-tRNA synthetase alpha chain	64.96%	2	7	37	137
IPI00283057.1	Isoform 2 of 5'-3' exoribonuclease 2	5'-3' exoribonuclease 2	48.47%	2	9	50	248
IPI00223060.1	phosphoenolpyruvate carboxykinase [GTP], mitochondrial	Phosphoenolpyruvate carboxykinase [GTP], mitochondrial	69.27%	3	17	59	468
IPI00313817.1	Hepatoma-derived growth factor	Hepatoma-derived growth factor	91.56%	2	12	30	430
IPI00319830.7	Isoform 1 of Spectrin beta chain, brain 1	Spectrin beta chain, brain 1	80.07%	2	13	246	1881
IPI01023212.1	Uncharacterized protein	Uncharacterized protein	60.27%	5	5	89	289
IPI00317710.1	Isoform 1 of Heat shock 70 kDa protein 4L	Heat shock 70 kDa protein 4L	81.03%	2	4	101	461
IPI00320399.7	Isoform Beta of Lamina-associated polypeptide 2, isoforms beta/delta/epsilon/gamma	Lamina-associated polypeptide 2, isoforms beta/delta/epsilon/gamma	88.50%	2	7	52	605
IPI00808098.1	Isoform 4 of Fatty acyl-CoA reductase 1	Fatty acyl-CoA reductase 1	75.34%	6	7	54	276
IPI00756696.2	Serine/threonine-protein phosphatase 4 regulatory subunit 2	Serine/threonine-protein phosphatase 4 regulatory subunit 2	87.29%	2	7	34	158
IPI00330862.5	Ezrin	Ezrin	82.42%	1	27	89	903
IPI00114209.1	Glutamate dehydrogenase 1, mitochondrial	Glutamate dehydrogenase 1, mitochondrial	93.19%	1	21	67	582
IPI00122289.4	E3 ubiquitin/ISG15 ligase TRIM25	E3 ubiquitin/ISG15 ligase TRIM25	76.03%	3	5	66	279
IPI00330523.1	Propionyl-CoA carboxylase alpha chain, mitochondrial	Propionyl-CoA carboxylase alpha chain, mitochondrial	70.17%	1	2	62	215
IPI00387337.1	Basic leucine zipper and W2 domain-containing protein 2	Basic leucine zipper and W2 domain-containing protein 2	79.95%	2	8	41	176
IPI00656325.2	Vesicle-fusing ATPase	Vesicle-fusing ATPase	77.28%	2	16	84	481
IPI00407339.7	Histone H4	Histone H4	76.70%	1	9	15	335
IPI00480250.2	Uncharacterized protein	Uncharacterized protein	58.48%	8	7	56	221
IPI00752412.1	Uncharacterized protein	Uncharacterized protein	68.09%	3	8	78	743
IPI00454049.4	Enoyl-CoA hydratase, mitochondrial	Enoyl-CoA hydratase, mitochondrial	75.52%	1	7	33	230
IPI00346834.1	Keratin, type II cytoskeletal 2 oral	Keratin, type II cytoskeletal 2 oral	79.29%	9	1	68	277
IPI00626132.3	Uncharacterized protein	Uncharacterized protein	83.03%	3	9	25	290
IPI00380331.1	Protein phosphatase 2A B56 delta subunit	Protein phosphatase 2A B56 delta subunit	61.51%	5	12	53	402
IPI00136498.1	Protein lin-7 homolog C	Lin-7 homolog C	55.84%	5	4	15	45
IPI00112963.1	Catenin alpha-1	Catenin alpha-1	84.99%	3	26	107	1015
IPI00270376.6	Isoform 1 of Coxsackievirus and adenovirus receptor homolog	Coxsackievirus and adenovirus receptor homolog	72.33%	2	3	24	149
IPI00955744.1	ribonuclease inhibitor isoform b	Ribonuclease inhibitor isoform b	98.58%	2	8	48	377
IPI00124096.1	Protein S100-A4	S100-A4	47.52%	1	4	12	123
IPI00919200.1	Uncharacterized protein	Uncharacterized protein	81.80%	5	10	49	234
IPI00121833.3	3-ketoacyl-CoA thiolase A, peroxisomal	3-ketoacyl-CoA thiolase A, peroxisomal	83.25%	3	16	35	435
IPI00221769.5	GTP:AMP phosphotransferase, mitochondrial	GTP:AMP phosphotransferase, mitochondrial	90.75%	1	12	29	271
IPI00649685.3	Isoform 1 of SWI/SNF-related matrix-associated actin-dependent regulator of chromatin subfamily D member 2	SWI/SNF-related matrix-associated actin-dependent regulator of chromatin subfamily D me	71.19%	2	3	49	161
IPI00338745.4	Non-histone chromosomal protein HMG-14	Non-histone chromosomal protein HMG-14	82.29%	3	8	13	1422
IPI00111770.7	ATP synthase subunit e, mitochondrial	ATP synthase subunit e, mitochondrial	98.59%	1	4	11	84
IPI00111821.6	Putative oxidoreductase GLYR1	Putative oxidoreductase GLYR1	67.95%	2	5	52	187
IPI00113517.1	Cathepsin B	Cathepsin B	80.83%	1	3	25	134
IPI00117689.1	Polymerase I and transcript release factor	Polymerase I and transcript release factor	67.35%	1	14	45	562
IPI00111218.1	Aldehyde dehydrogenase, mitochondrial	Aldehyde dehydrogenase, mitochondrial	77.46%	1	11	45	367
IPI00125298.2	Isoform p66Shc of SHC-transforming protein 1	SHC-transforming protein 1	56.99%	3	4	42	258
IPI00653717.3	Putative uncharacterized protein (Fragment)	Putative uncharacterized protein (Fragment)	72.27%	1	1	70	377
IPI00420569.2	Sodium/potassium-transporting ATPase subunit alpha-2	Sodium/potassium-transporting ATPase subunit alpha-2	70.88%	3	3	84	643
IPI00121788.1	Peroxiredoxin-1	Peroxiredoxin-1	86.43%	6	16	27	461
IPI00457578.2	Isoform 1 of Villin-like protein	Villin-like protein	59.84%	4	7	58	186
IPI00132722.9	Annexin A3	Annexin A3	92.88%	2	21	51	531
IPI00228385.5	Glucose-6-phosphate 1-dehydrogenase X	Glucose-6-phosphate 1-dehydrogenase X	61.55%	3	12	43	302
IPI00990529.1	Uncharacterized protein (Fragment)	Uncharacterized protein (Fragment)	96.65%	3	12	19	361
IPI00130322.5	NADH dehydrogenase [ubiquinone] 1 alpha subcomplex subunit 7	NADH dehydrogenase [ubiquinone] 1 alpha subcomplex subunit 7	91.15%	1	4	21	148
IPI00311682.5	Sodium/potassium-transporting ATPase subunit alpha-1	Sodium/potassium-transporting ATPase subunit alpha-1	61.68%	3	20	82	1144
IPI00318614.9	Isocitrate dehydrogenase [NADP], mitochondrial	Isocitrate dehydrogenase [NADP], mitochondrial	97.12%	4	18	61	439
IPI00119115.2	V-type proton ATPase subunit E 1	V-type proton ATPase subunit E 1	83.19%	2	5	35	142
IPI00109212.3	Sorting nexin-2	Sorting nexin-2	72.83%	1	11	54	314
IPI00132799.4	complement component 1 Q subcomponent-binding protein, mitochondrial	Complement component 1 Q subcomponent-binding protein, mitochondrial	71.68%	2	5	20	251
IPI00131176.1	Cytochrome c oxidase subunit 2	Cytochrome c oxidase subunit 2	44.93%	1	4	7	123
IPI00331146.5	UMP-CMP kinase	UMP-CMP kinase	64.76%	1	8	22	228
IPI00331182.4	Uncharacterized protein	Uncharacterized protein	84.03%	1	20	68	409
IPI00135231.2	Isocitrate dehydrogenase	Isocitrate dehydrogenase	89.34%	2	20	62	371
IPI00808370.2	Protein diaphanous homolog 1	Diaphanous homolog 1	67.17%	2	18	112	438
IPI00284806.8	Hypoxanthine-guanine phosphoribosyltransferase	Hypoxanthine-guanine phosphoribosyltransferase	70.18%	1	6	20	125
IPI00329942.4	Isoform 2 of Vacuolar protein sorting-associated protein 26A	Vacuolar protein sorting-associated protein 26A	86.35%	3	6	40	178
IPI00230277.3	Mitogen-activated protein kinase 3	Mitogen-activated protein kinase 3	69.47%	13	4	32	157
IPI00169958.3	Lanosterol synthase	Lanosterol synthase	53.62%	1	3	38	113
IPI00229475.1	Junction plakoglobin	Junction plakoglobin	78.52%	1	17	74	547
IPI00849478.1	phosphoserine aminotransferase-like isoform 2	phosphoserine aminotransferase-like isoform 2	92.16%	2	20	45	652
IPI00116753.4	Electron transfer flavoprotein subunit alpha, mitochondrial	Electron transfer flavoprotein subunit alpha, mitochondrial	92.79%	1	13	36	475
IPI00467833.6	triosephosphate isomerase	Triosephosphate isomerase	95.65%	2	16	33	632
IPI00313968.8	Isoform 1 of Histone-lysine N-methyltransferase setd3	Histone-lysine N-methyltransferase setd3	66.16%	3	5	48	209
IPI00121440.4	Electron transfer flavoprotein subunit beta	Electron transfer flavoprotein subunit beta	89.02%	1	11	25	136
IPI00312058.6	Catalase	Catalase	80.46%	1	9	46	194
IPI00553419.3	Desmoplakin	Desmoplakin	80.06%	2	28	362	1460
IPI00134344.6	Putative uncharacterized protein	Putative uncharacterized protein	71.11%	2	1	197	608
IPI00751369.1	L-lactate dehydrogenase A chain isoform 2	L-lactate dehydrogenase A chain isoform 2	96.68%	5	20	51	781
IPI00461514.4	Histone H2B	Histone H2B	90.37%	21	11	27	626
IPI00986206.1	Uncharacterized protein	Uncharacterized protein	74.29%	2	3	15	211
IPI00989397.1	src substrate cortactin	Src substrate cortactin	64.95%	2	3	40	487
IPI00113389.5	Protein Niban	Niban	61.34%	2	17	56	299
IPI00229510.5	L-lactate dehydrogenase B chain	L-lactate dehydrogenase B chain	75.15%	6	4	34	147
IPI00135324.2	solute carrier family 12 member 2	Solute carrier family 12 member 2	56.05%	2	23	73	414
IPI00123278.1	Pyrroline-5-carboxylate reductase 2	Pyrroline-5-carboxylate reductase 2	89.06%	1	6	31	236
IPI00228253.2	Acetyl-CoA acetyltransferase, cytosolic	Acetyl-CoA acetyltransferase, cytosolic	79.60%	4	8	32	198
IPI00129395.2	Large neutral amino acids transporter small subunit 1	Large neutral amino acids transporter small subunit 1	42.58%	1	4	19	153
IPI00989544.2	Uncharacterized protein (Fragment)	Uncharacterized protein (Fragment)	68.36%	4	11	18	267
IPI00331692.1	enoyl-CoA delta isomerase 1, mitochondrial precursor	Enoyl-CoA delta isomerase 1, mitochondrial precursor	69.90%	1	7	28	174
IPI00742310.5	RNA-binding protein EWS	RNA-binding protein EWS	39.24%	4	3	40	282
IPI00125899.1	Catenin beta-1	Catenin beta-1	79.90%	3	12	63	327
IPI00649157.1	Stathmin	Stathmin	80.27%	1	1	19	130
IPI00121550.3	Sodium/potassium-transporting ATPase subunit beta-1	Sodium/potassium-transporting ATPase subunit beta-1	64.47%	1	7	23	138
IPI00222386.3	Charged multivesicular body protein 2b	Charged multivesicular body protein 2b	60.56%	1	5	26	162
IPI00467914.3	Histone H1.0	Histone H1.0	83.51%	8	10	37	287
IPI00137409.3	Transketolase	Transketolase	93.90%	1	30	68	1309
IPI00121427.1	Protein S100-A6	S100-A6	97.75%	1	10	15	353
IPI00457485.3	Isoform 2 of Protein LAP2	Protein LAP2	75.52%	5	6	117	458
IPI00282848.1	20 kDa protein	20 kDa protein	87.85%	2	1	32	311
IPI00320188.5	Nicotinamide phosphoribosyltransferase	Nicotinamide phosphoribosyltransferase	76.99%	1	7	40	110
IPI00230264.5	Histone H2A.x	Histone H2A.x	95.80%	1	8	25	459
IPI00551236.3	Stathmin	Stathmin	93.29%	3	3	29	282
IPI00135646.2	ATP-binding cassette sub-family D member 3	ATP-binding cassette sub-family D member 3	75.11%	1	6	67	266
IPI00553294.1	Uncharacterized protein	Uncharacterized protein	71.74%	9	1	13	627
IPI00331734.5	Histone H2A.Z	Histone H2A.Z	88.28%	5	4	17	99
IPI00319556.6	histone H1t	Histone H1t	70.81%	1	1	33	142
IPI00115492.1	Isoform 1 of Epidermal growth factor receptor kinase substrate 8-like protein 2	Epidermal growth factor receptor kinase substrate 8-like protein 2	61.59%	2	10	60	272
IPI00268688.4	CD2-associated protein	CD2-associated protein	76.92%	1	18	54	338
IPI00553538.3	Histone H3.1	Histone H3.1	77.21%	1	1	19	159
IPI00230133.5	Histone H1.5	Histone H1.5	86.55%	1	15	50	742
IPI00230113.5	Cytochrome b5	Cytochrome b5	88.81%	3	5	12	140
IPI00223714.5	Histone H1.4	Histone H1.4	86.76%	8	7	52	754
IPI00331597.6	Histone H1.3	Histone H1.3	90.95%	2	3	55	821
IPI00223713.5	Histone H1.2	Histone H1.2	87.26%	1	6	49	632
IPI00227140.1	Keratin, type I cytoskeletal 14	Keratin, type I cytoskeletal 14	79.75%	10	7	61	410
IPI00170101.1	Optineurin	Optineurin	92.29%	1	13	75	299
IPI00128857.2	NADP-dependent malic enzyme	NADP-dependent malic enzyme	92.83%	3	21	59	446
IPI00319270.2	Perilipin-3	Perilipin-3	91.99%	1	12	44	452
IPI00137194.1	Monocarboxylate transporter 1	Monocarboxylate transporter 1	41.58%	1	6	17	205
IPI00112947.1	Keratin, type I cytoskeletal 19	Keratin, type I cytoskeletal 19	97.27%	5	28	66	1088
IPI00353727.8	Annexin A4	Annexin A4	90.60%	2	19	47	429
IPI00134961.1	Medium-chain specific acyl-CoA dehydrogenase, mitochondrial	Medium-chain specific acyl-CoA dehydrogenase, mitochondrial	72.45%	2	4	37	126
IPI00311626.5	High mobility group nucleosome-binding domain-containing protein 5	High mobility group nucleosome-binding domain-containing protein 5	57.39%	1	8	31	132
IPI00123276.1	MOSC domain-containing protein 2, mitochondrial	MOSC domain-containing protein 2, mitochondrial	72.78%	4	5	28	119
IPI00131933.1	Claudin-2	Claudin-2	51.74%	1	3	9	149
IPI00468696.3	Keratin, type I cytoskeletal 42	Keratin, type I cytoskeletal 42	84.29%	5	1	56	468
IPI00109311.3	Isoform 1 of Na(+)/H(+) exchange regulatory cofactor NHE-RF1	Na(+)/H(+) exchange regulatory cofactor NHE-RF1	84.79%	1	11	39	279
