# Supplementary material for: The Prion Protein Controls Polysialylation of Neural Cell Adhesion Molecule 1 during Cellular Morphogenesis
Source: PLoS One. 2015 Aug 19;10(8):e0133741. doi: 10.1371/journal.pone.0133741 (PMC4546001; doi:10.1371/journal.pone.0133741)
Supplement: S2 Table — (PDF) [file pone.0133741.s004.pdf]

S2 Table: Top 200 proteins exhibiting most pronounced differences in expression before and after 48 hours TGFB1 treatment in wt NMuMG cells (extracted from dataset I)

Accession	Original database description	Modified description
IPI00131547.1	Plasminogen activator inhibitor 1	Plasminogen activator inhibitor 1
IPI00856302.1	Isoform 6 of Palladin	Palladin
IPI00134746.5	Argininosuccinate synthase	Argininosuccinate synthase
IPI00124513.1	LIM and cysteine-rich domains protein 1	LIM and cysteine-rich domains protein 1
IPI00230665.3	neural cell adhesion molecule 1 isoform 3	Neural cell adhesion molecule 1 isoform 3
IPI00331612.3	High mobility group protein HMGI-C	High mobility group protein HMGI-C
IPI00122257.1	5'-nucleotidase	5'-nucleotidase
IPI00265705.2	Isoform 2 of Mitogen-activated protein kinase kinase kinase MLT	Mitogen-activated protein kinase kinase kinase MLT
IPI00128209.3	Isoform 1 of Adenylate kinase isoenzyme 1	Adenylate kinase isoenzyme 1
IPI00608020.4	ferritin light chain 1	Ferritin light chain 1
IPI00320420.3	Clusterin	Clusterin
IPI00281011.7	MARCKS-related protein	MARCKS-related protein
IPI00670967.3	Fermitin family homolog 1	Fermitin family homolog 1
IPI00277930.7	Putative uncharacterized protein	Putative uncharacterized protein
IPI00970075.1	Uncharacterized protein	Uncharacterized protein
IPI00387370.1	Isoform AGX2 of UDP-N-acetylhexosamine pyrophosphorylase	UDP-N-acetylhexosamine pyrophosphorylase
IPI00275753.1	Scr3	Scr3
IPI00123758.3	procollagen-lysine,2-oxoglutarate 5-dioxygenase 2 isoform 1	Procollagen-lysine,2-oxoglutarate 5-dioxygenase 2 isoform 1
IPI00114342.1	Hexokinase-2	Hexokinase-2
IPI00120245.2	Integrin alpha-V	Integrin alpha-V
IPI00123540.1	Tetraspanin-7	Tetraspanin-7
IPI00130240.1	Peptidyl-prolyl cis-trans isomerase C	Peptidyl-prolyl cis-trans isomerase C
IPI00399959.1	Isoform 2 of Prolyl 4-hydroxylase subunit alpha-1	Prolyl 4-hydroxylase subunit alpha-1
IPI00756703.1	Isoform 2 of Serine/threonine-protein phosphatase 2B catalytic subunit alpha isoform	Serine/threonine-protein phosphatase 2B catalytic subunit
IPI00830803.1	fibulin-2 isoform b	Fibulin-2 isoform b
IPI00331598.2	Uncharacterized protein	Uncharacterized protein
IPI00308785.1	Prostaglandin G/H synthase 2	Prostaglandin G/H synthase 2
IPI00268377.7	Beta-parvin	Beta-parvin
IPI00555103.2	protein MICAL-2 isoform A	MICAL-2 isoform A
IPI00132474.3	Integrin beta-1	Integrin beta-1
IPI00330289.4	Uncharacterized protein	Uncharacterized protein
IPI00990293.1	band 4.1-like protein 2	Band 4.1-like protein 2
IPI00124803.1	Zinc finger protein ZPR1	Zinc finger protein ZPR1
IPI00118413.2	Uncharacterized protein	Uncharacterized protein
IPI00123202.1	Ataxin-10	Ataxin-10
IPI00320480.3	Sphingosine-1-phosphate lyase 1	Sphingosine-1-phosphate lyase 1
IPI00133608.3	Isoform 1 of OCIA domain-containing protein 1	OCIA domain-containing protein 1
IPI00223047.2	Cytoskeleton-associated protein 4	Cytoskeleton-associated protein 4
IPI00457611.2	Isoform 1 of Septin-9	Septin-9
IPI00114733.2	Serpin H1	Serpin H1
IPI00654388.2	Isoform 1 of Leucine-rich repeat flightless-interacting protein 1	Leucine-rich repeat flightless-interacting protein 1
IPI00395177.1	active breakpoint cluster region-related protein isoform 2	active breakpoint cluster region-related protein isoform 2
IPI00126892.12	Putative uncharacterized protein	Putative uncharacterized protein
IPI00652210.1	Putative uncharacterized protein (Fragment)	Putative uncharacterized protein (Fragment)
IPI00228757.1	Isoform Short of Thymosin beta-4	Thymosin beta-4
IPI00119776.1	Isoform 1 of Something about silencing protein 10	Something about silencing protein 10
IPI00221632.3	Isoform 1 of Y+L amino acid transporter 2	Y+L amino acid transporter 2
IPI00671957.2	glutaminase isoform 2	Glutaminase isoform 2
IPI00229801.5	tumor suppressor p53-binding protein 1	Tumor suppressor p53-binding protein 1
IPI00775950.1	Uncharacterized protein	Uncharacterized protein
IPI00274639.4	Isoform 1 of Mitogen-activated protein kinase kinase kinase MLT	Mitogen-activated protein kinase kinase kinase MLT
IPI00322749.3	Small nuclear ribonucleoprotein Sm D1	Small nuclear ribonucleoprotein Sm D1
IPI00975193.1	Putative uncharacterized protein	Putative uncharacterized protein
IPI00119111.2	Calponin-3	Calponin-3
IPI00116077.3	Transmembrane protein 111	Transmembrane protein 111
IPI00135475.3	Isoform A of Drebrin	Drebrin
IPI00109044.8	myosin light chain, regulatory B-like	Myosin light chain, regulatory B-like
IPI00169870.6	Procollagen galactosyltransferase 1	Procollagen galactosyltransferase 1
IPI00458777.2	myc box-dependent-interacting protein 1 isoform 2	Myc box-dependent-interacting protein 1 isoform 2
IPI00409176.1	Isoform 1 of Calpastatin	Calpastatin
IPI00119760.4	Nucleolar protein 10	Nucleolar protein 10
IPI00137166.1	Transcription factor jun-B	Transcription factor jun-B
IPI00112072.1	Isoform 1 of Minor histocompatibility antigen H13	Minor histocompatibility antigen H13
IPI00228360.4	Zyxin	Zyxin
IPI00123181.4	Myosin-9	Myosin-9
IPI00308938.6	Calpain-2 catalytic subunit	Calpain-2 catalytic subunit
IPI00317309.5	Annexin A5	Annexin A5
IPI00465786.4	Talin-1	Talin-1
IPI00453692.4	Isoform 1 of Nestin	Nestin
IPI00462232.3	myosin light polypeptide 6-like	Myosin light polypeptide 6-like
IPI00751474.1	plasma membrane calcium ATPase 4 isoform x/e	Plasma membrane calcium ATPase 4 isoform x/e
IPI00110588.4	Moesin	Moesin
IPI00380436.1	Alpha-actinin-1	Alpha-actinin-1
IPI00458057.3	Testis-expressed sequence 10 protein	Testis-expressed sequence 10 protein
IPI00474959.2	Putative uncharacterized protein	Putative uncharacterized protein
IPI00462502.3	Isoform 1 of Protein LAS1 homolog	Protein LAS1 homolog
IPI00467104.2	Protein flightless-1 homolog	Flightless-1 homolog
IPI00229647.6	talin-2	Talin-2
IPI00153234.2	Pyrroline-5-carboxylate reductase 3	Pyrroline-5-carboxylate reductase 3
IPI00119632.3	Putative rRNA methyltransferase 3	Putative rRNA methyltransferase 3
IPI00263313.1	Developmentally-regulated GTP-binding protein 1	Developmentally-regulated GTP-binding protein 1
IPI00125778.4	Transgelin-2	Transgelin-2
IPI00986743.1	Microtubule-associated protein	Microtubule-associated protein
IPI00420870.4	Isoform 1 of UDP-N-acetylglucosamine--peptide N-acetylglucosaminyltransferase 110 kDa subunit	UDP-N-acetylglucosamine--peptide N-acetylglucosaminyltransferase 110 kDa subu
IPI00222675.3	D19Bwg1357e protein	D19Bwg1357e protein
IPI00230642.1	Isoform 3 of Calpastatin	Calpastatin
IPI00311453.5	putative ribosomal RNA methyltransferase NOP2	putative ribosomal RNA methyltransferase NOP2
IPI00126913.5	Isoform 1 of ATPase family AAA domain-containing protein 3	ATPase family AAA domain-containing protein 3
IPI00128071.7	Isoform 1 of General vesicular transport factor p115	General vesicular transport factor p115
IPI00116649.1	Calponin-2	Calponin-2
IPI00129517.1	Isoform Mitochondrial of Peroxiredoxin-5, mitochondrial	Peroxiredoxin-5, mitochondrial
IPI00311344.4	Uncharacterized protein	Uncharacterized protein
IPI00309768.7	PDZ and LIM domain protein 1	PDZ and LIM domain protein 1
IPI00122220.1	RNA 3'-terminal phosphate cyclase-like protein	RNA 3'-terminal phosphate cyclase-like protein
IPI00420344.1	RRP12-like protein	RRP12-like protein
IPI00336958.3	Isoform 1 of Zinc finger CCCH domain-containing protein 15	Zinc finger CCCH domain-containing protein 15
IPI00985577.1	Uncharacterized protein	Uncharacterized protein
IPI00331556.5	Heat shock 70 kDa protein 4	Heat shock 70 kDa protein 4
IPI00130607.8	Isoform 1 of PDZ and LIM domain protein 7	PDZ and LIM domain protein 7
IPI00126077.2	Integrin alpha-2	Integrin alpha-2
IPI00346834.1	Keratin, type II cytoskeletal 2 oral	Keratin, type II cytoskeletal 2 oral
IPI00626132.3	Uncharacterized protein	Uncharacterized protein
IPI00380331.1	Protein phosphatase 2A B56 delta subunit	Protein phosphatase 2A B56 delta subunit
IPI00136498.1	Protein lin-7 homolog C	Lin-7 homolog C
IPI00112963.1	Catenin alpha-1	Catenin alpha-1
IPI00270376.6	Isoform 1 of Coxsackievirus and adenovirus receptor homolog	Coxsackievirus and adenovirus receptor homolog
IPI00955744.1	ribonuclease inhibitor isoform b	Ribonuclease inhibitor isoform b
IPI00124096.1	Protein S100-A4	S100-A4
IPI00919200.1	Uncharacterized protein	Uncharacterized protein
IPI00121833.3	3-ketoacyl-CoA thiolase A, peroxisomal	3-ketoacyl-CoA thiolase A, peroxisomal
IPI00221769.5	GTP:AMP phosphotransferase, mitochondrial	GTP:AMP phosphotransferase, mitochondrial
IPI00649685.3	Isoform 1 of SWI/SNF-related matrix-associated actin-dependent regulator of chromatin subfamily D member 2	SWI/SNF-related matrix-associated actin-dependent regulator
IPI00338745.4	Non-histone chromosomal protein HMG-14	Non-histone chromosomal protein HMG-14
IPI00111770.7	ATP synthase subunit e, mitochondrial	ATP synthase subunit e, mitochondrial
IPI00111821.6	Putative oxidoreductase GLYR1	Putative oxidoreductase GLYR1
IPI00113517.1	Cathepsin B	Cathepsin B
IPI00117689.1	Polymerase I and transcript release factor	Polymerase I and transcript release factor
IPI00111218.1	Aldehyde dehydrogenase, mitochondrial	Aldehyde dehydrogenase, mitochondrial
IPI00125298.2	Isoform p66Shc of SHC-transforming protein 1	SHC-transforming protein 1
IPI00653717.3	Putative uncharacterized protein (Fragment)	Putative uncharacterized protein (Fragment)
IPI00420569.2	Sodium/potassium-transporting ATPase subunit alpha-2	Sodium/potassium-transporting ATPase subunit alpha-2
IPI00121788.1	Peroxiredoxin-1	Peroxiredoxin-1
IPI00457578.2	Isoform 1 of Villin-like protein	Villin-like protein
IPI00132722.9	Annexin A3	Annexin A3
IPI00228385.5	Glucose-6-phosphate 1-dehydrogenase X	Glucose-6-phosphate 1-dehydrogenase X
IPI00990529.1	Uncharacterized protein (Fragment)	Uncharacterized protein (Fragment)
IPI00130322.5	NADH dehydrogenase [ubiquinone] 1 alpha subcomplex subunit 7	NADH dehydrogenase [ubiquinone] 1 alpha subcomplex subunit 7
IPI00311682.5	Sodium/potassium-transporting ATPase subunit alpha-1	Sodium/potassium-transporting ATPase subunit alpha-1
IPI00318614.9	Isocitrate dehydrogenase [NADP], mitochondrial	Isocitrate dehydrogenase [NADP], mitochondrial
IPI00119115.2	V-type proton ATPase subunit E 1	V-type proton ATPase subunit E 1
IPI00109212.3	Sorting nexin-2	Sorting nexin-2
IPI00132799.4	complement component 1 Q subcomponent-binding protein, mitochondria	Complement component 1 Q subcomponent-binding protein
IPI00131176.1	Cytochrome c oxidase subunit 2	Cytochrome c oxidase subunit 2
IPI00331146.5	UMP-CMP kinase	UMP-CMP kinase
IPI00331182.4	Uncharacterized protein	Uncharacterized protein
IPI00135231.2	Isocitrate dehydrogenase	Isocitrate dehydrogenase
IPI00808370.2	Protein diaphanous homolog 1	Diaphanous homolog 1
IPI00284806.8	Hypoxanthine-guanine phosphoribosyltransferase	Hypoxanthine-guanine phosphoribosyltransferase
IPI00329942.4	Isoform 2 of Vacuolar protein sorting-associated protein 26A	Vacuolar protein sorting-associated protein 26A
IPI00230277.3	Mitogen-activated protein kinase 3	Mitogen-activated protein kinase 3
IPI00169958.3	Lanosterol synthase	Lanosterol synthase
IPI00229475.1	Junction plakoglobin	Junction plakoglobin
IPI00849478.1	phosphoserine aminotransferase-like isoform 2	phosphoserine aminotransferase-like isoform 2
IPI00116753.4	Electron transfer flavoprotein subunit alpha, mitochondrial	Electron transfer flavoprotein subunit alpha, mitochondrial
IPI00467833.6	triosephosphate isomerase	Triosephosphate isomerase
IPI00313968.8	Isoform 1 of Histone-lysine N-methyltransferase setd3	Histone-lysine N-methyltransferase setd3
IPI00121440.4	Electron transfer flavoprotein subunit beta	Electron transfer flavoprotein subunit beta
IPI00312058.6	Catalase	Catalase
IPI00553419.3	Desmoplakin	Desmoplakin
IPI00134344.6	Putative uncharacterized protein	Putative uncharacterized protein
IPI00751369.1	L-lactate dehydrogenase A chain isoform 2	L-lactate dehydrogenase A chain isoform 2
IPI00461514.4	Histone H2B	Histone H2B
IPI00986206.1	Uncharacterized protein	Uncharacterized protein
IPI00989397.1	src substrate cortactin	Src substrate cortactin
IPI00113389.5	Protein Niban	Niban
IPI00229510.5	L-lactate dehydrogenase B chain	L-lactate dehydrogenase B chain
IPI00135324.2	solute carrier family 12 member 2	Solute carrier family 12 member 2
IPI00123278.1	Pyrroline-5-carboxylate reductase 2	Pyrroline-5-carboxylate reductase 2
IPI00228253.2	Acetyl-CoA acetyltransferase, cytosolic	Acetyl-CoA acetyltransferase, cytosolic
IPI00129395.2	Large neutral amino acids transporter small subunit 1	Large neutral amino acids transporter small subunit 1
IPI00989544.2	Uncharacterized protein (Fragment)	Uncharacterized protein (Fragment)
IPI00331692.1	enoyl-CoA delta isomerase 1, mitochondrial precursor	Enoyl-CoA delta isomerase 1, mitochondrial precursor
IPI00742310.5	RNA-binding protein EWS	RNA-binding protein EWS
IPI00125899.1	Catenin beta-1	Catenin beta-1
IPI00649157.1	Stathmin	Stathmin
IPI00121550.3	Sodium/potassium-transporting ATPase subunit beta-1	Sodium/potassium-transporting ATPase subunit beta-1
IPI00222386.3	Charged multivesicular body protein 2b	Charged multivesicular body protein 2b
IPI00467914.3	Histone H1.0	Histone H1.0
IPI00137409.3	Transketolase	Transketolase
IPI00121427.1	Protein S100-A6	S100-A6
IPI00457485.3	Isoform 2 of Protein LAP2	Protein LAP2
IPI00282848.1	20 kDa protein	20 kDa protein
IPI00320188.5	Nicotinamide phosphoribosyltransferase	Nicotinamide phosphoribosyltransferase
IPI00230264.5	Histone H2A.x	Histone H2A.x
IPI00551236.3	Stathmin	Stathmin
IPI00135646.2	ATP-binding cassette sub-family D member 3	ATP-binding cassette sub-family D member 3
IPI00553294.1	Uncharacterized protein	Uncharacterized protein
IPI00331734.5	Histone H2A.Z	Histone H2A.Z
IPI00319556.6	histone H1t	Histone H1t
IPI00115492.1	Isoform 1 of Epidermal growth factor receptor kinase substrate 8-like protein 2	Epidermal growth factor receptor kinase substrate 8-like protein 2
IPI00268688.4	CD2-associated protein	CD2-associated protein
IPI00553538.3	Histone H3.1	Histone H3.1
IPI00230133.5	Histone H1.5	Histone H1.5
IPI00230113.5	Cytochrome b5	Cytochrome b5
IPI00223714.5	Histone H1.4	Histone H1.4
IPI00331597.6	Histone H1.3	Histone H1.3
IPI00223713.5	Histone H1.2	Histone H1.2
IPI00227140.1	Keratin, type I cytoskeletal 14	Keratin, type I cytoskeletal 14
IPI00170101.1	Optineurin	Optineurin
IPI00128857.2	NADP-dependent malic enzyme	NADP-dependent malic enzyme
IPI00319270.2	Perilipin-3	Perilipin-3
IPI00137194.1	Monocarboxylate transporter 1	Monocarboxylate transporter 1
IPI00112947.1	Keratin, type I cytoskeletal 19	Keratin, type I cytoskeletal 19
IPI00353727.8	Annexin A4	Annexin A4
IPI00134961.1	Medium-chain specific acyl-CoA dehydrogenase, mitochondrial	Medium-chain specific acyl-CoA dehydrogenase, mitochondrial
IPI00311626.5	High mobility group nucleosome-binding domain-containing protein 5	High mobility group nucleosome-binding domain-containing protein 5
IPI00123276.1	MOSC domain-containing protein 2, mitochondrial	MOSC domain-containing protein 2, mitochondrial
IPI00131933.1	Claudin-2	Claudin-2
IPI00468696.3	Keratin, type I cytoskeletal 42	Keratin, type I cytoskeletal 42
IPI00109311.3	Isoform 1 of Na(+)/H(+) exchange regulatory cofactor NHE-RF1	Na(+)/H(+) exchange regulatory cofactor NHE-RF1

Accession	Coverage	# Proteins	# Unique Peptides	# Peptides	# PSMs	TMT Ratios 126/131	Count	Variability [%]	127/131	Count	Variability [%]	128/131	Count	Variability [%]	129/131	Count	Variability [%]	130/131	Count	Variability [%]	# Peptides Sequest	# Peptides Mascot	# PSM Sequest	# PSM Mascot	Coverage Sequest	Coverage Mascot	Score Sequest	Score Mascot	# AAs	MW [kDa]	pI	Averages
IPI00131547.1	82.84%	1	10	35	262	0.159	5	230.1	1.032	5	8.7	0.206	5	72.3	1.013	5	14.5	0.267	5	122	12	13	30	40	39.05%	33.33%	28.77	51.39	402	45.1	6.64	0.211
IPI00856302.1	61.90%	7	9	91	515	0.282	12	75.5	0.938	13	9.9	0.207	12	93	0.911	13	21.4	0.353	13	65.7	19	26	41	55	16.53%	26.60%	57.5	48.63	1391	150.5	6.24	0.281
IPI00134746.5	77.67%	1	17	45	458	0.254	19	170.9	1.024	19	4.8	0.34	19	143.3	1.051	19	7.9	0.353	19	138.4	19	17	36	51	43.20%	44.17%	42.45	31.64	412	46.6	8.22	0.316
IPI00124513.1	73.70%	1	5	32	158	0.346	4	61.9	1.068	4	19.6	0.339	4	48.6	1.143	4	4.3	0.409	4	45.4	9	11	12	22	35.89%	36.44%	17.83	68.83	365	41.0	7.93	0.365
IPI00230665.3	73.27%	4	14	84	731	0.367	29	29.3	0.989	29	10.3	0.353	29	25.9	0.943	29	12.8	0.378	28	21	32	24	76	66	31.48%	31.84%	122.49	89.18	1115	119.2	4.82	0.366
IPI00331612.3	75.93%	2	5	11	452	0.411	25	52.8	0.971	26	16.3	0.453	26	61.4	1.016	26	15.3	0.317	24	122.1	6	8	39	64	53.70%	68.52%	97.77	597.35	108	11.8	10.62	0.394
IPI00122257.1	71.01%	2	7	43	175	0.375	7	77.4	1.009	7	12.8	0.426	7	83.3	0.906	7	20.6	0.388	7	68.9	7	8	17	19	15.80%	14.93%	34.33	61.4	576	63.8	6.64	0.396
IPI00265705.2	36.56%	1	2	19	104	0.51	7	50.3	0.932	7	9.5	0.34	7	30.8	0.872	7	23.3	0.357	7	15.2	7	7	15	16	15.86%	17.40%	22.35	77.78	454	51.3	5.35	0.402
IPI00128209.3	91.75%	3	6	22	124	0.44	15	65.8	1.059	15	7.2	0.411	14	67.7	1.041	15	5.2	0.449	15	77.4	10	9	24	20	48.45%	56.70%	43.61	131.64	194	21.5	5.81	0.433
IPI00608020.4	85.25%	3	5	21	301	0.432	16	38.8	0.998	19	25.4	0.449	17	62.3	1.095	18	16.2	0.445	18	48.4	10	7	32	35	64.48%	48.09%	58.17	136.93	183	20.8	6.00	0.442
IPI00320420.3	62.28%	1	10	39	249	0.382	16	83.2	0.983	16	9.9	0.471	16	66.6	0.952	16	8	0.49	16	87.5	9	14	29	34	20.98%	29.46%	42.92	50.64	448	51.6	5.67	0.448
IPI00281011.7	91.50%	1	8	13	297	0.456	19	53.8	1.005	25	13	0.483	21	52.9	0.977	25	18.5	0.453	19	152.5	8	6	38	36	60.50%	54.00%	99.74	327.96	200	20.2	4.61	0.464
IPI00670967.3	70.90%	2	4	47	171	0.512	5	32.5	0.933	5	5.3	0.402	5	137.1	0.962	5	14.3	0.509	4	53	5	16	13	36	9.16%	28.95%	12.79	49.09	677	76.9	6.27	0.474
IPI00277930.7 … IPI00109311.3 (rows 14–200)	[illegible]	[illegible]	[illegible]	[illegible]	[illegible]	[illegible]	[illegible]	[illegible]	[illegible]	[illegible]	[illegible]	[illegible]	[illegible]	[illegible]	[illegible]	[illegible]	[illegible]	[illegible]	[illegible]	[illegible]	[illegible]	[illegible]	[illegible]	[illegible]	[illegible]	[illegible]	[illegible]	[illegible]	[illegible]	[illegible]	[illegible]	[illegible]	[illegible]
