# Supplementary material for: The Prion Protein Controls Polysialylation of Neural Cell Adhesion Molecule 1 during Cellular Morphogenesis
Source: PLoS One. 2015 Aug 19;10(8):e0133741. doi: 10.1371/journal.pone.0133741 (PMC4546001; doi:10.1371/journal.pone.0133741)
Supplement: S4 Table — (PDF) [file pone.0133741.s006.pdf]

S4 Table: Overlap of top 200 proteins undergoing the most pronounced level changes during 48 hour TGFB1 treatment (dataset I) AND top 200 proteins observed at most divergent levels in a direct comparison of 48 hour TGFB1-treated stable PrP kd and wild-type cells (dataset II)

						TMT Ratios											Averages	
						I				II							I	II
						-/+TGFB1				stable v wt (+TGFB1)					MW			
Accession	Description	Coverage	# Proteins	# Unique Peptides	# Peptides	126/131	128/131	130/131	Count	126/131	128/131	130/131	Count	# AAs	[kDa]	pI	-/+TGFB1	stable kd v. wt
IPI00856302.1	Palladin	61.90%	7	9	91	0.282	0.207	0.353	13	0.828	0.849	0.739	19	1391	150.5	6.24	0.281	0.805
IPI00134746.5	Argininosuccinate synthase	77.67%	1	17	45	0.254	0.340	0.353	19	0.859	0.898	0.874	45	412	46.6	8.22	0.316	0.877
IPI00124513.1	LIM and cysteine-rich domains protein 1	73.70%	1	5	32	0.346	0.339	0.409	4	0.671	0.696	0.714	9	365	41.0	7.93	0.365	0.694
IPI00230665.3	Neural cell adhesion molecule 1 isoform 3	73.27%	4	14	84	0.367	0.353	0.378	28	0.762	0.757	0.751	57	1115	119.2	4.82	0.366	0.757
IPI00331612.3	High mobility group protein HMGI-C	75.93%	2	5	11	0.411	0.453	0.317	24	1.195	1.111	1.120	39	108	11.8	10.62	0.394	1.142
IPI00122257.1	5'-nucleotidase	71.01%	2	7	43	0.375	0.426	0.388	7	0.374	0.373	0.375	7	576	63.8	6.64	0.396	0.374
IPI00608020.4	Ferritin light chain 1	85.25%	3	5	21	0.432	0.449	0.445	18	0.592	0.607	0.596	26	183	20.8	6.00	0.442	0.598
IPI00320420.3	Clusterin	62.28%	1	10	39	0.382	0.471	0.490	16	0.741	0.818	0.815	22	448	51.6	5.67	0.448	0.791
IPI00281011.7	MARCKS-related protein	91.50%	1	8	13	0.456	0.483	0.453	19	0.736	0.789	0.756	26	200	20.2	4.61	0.464	0.760
IPI00670967.3	Fermitin family homolog 1	70.90%	2	4	47	0.512	0.402	0.509	4	0.817	1.015	0.792	6	677	76.9	6.27	0.474	0.875
IPI00387370.1	UDP-N-acetylhexosamine pyrophosphorylase	58.81%	5	9	36	0.548	0.495	0.438	4	0.773	0.808	0.801	9	522	58.6	6.49	0.494	0.794
IPI00120245.2	Integrin alpha-V	70.21%	2	14	85	0.584	0.578	0.562	22	0.859	0.848	0.814	35	1044	115.3	5.63	0.575	0.840
IPI00130240.1	Peptidyl-prolyl cis-trans isomerase C	69.81%	1	3	14	0.714	0.560	0.481	4	1.263	1.275	1.331	11	212	22.8	7.50	0.585	1.290
IPI00756703.1	Serine/threonine-protein phosphatase 2B	74.95%	12	6	40	0.563	0.591	0.637	4	1.159	1.173	1.209	11	511	57.6	6.30	0.597	1.180
IPI00830803.1	Fibulin-2 isoform b	56.05%	1	9	49	0.545	0.707	0.563	14	0.842	0.892	0.828	26	1174	126.4	4.65	0.605	0.854
IPI00555103.2	MICAL-2 isoform A	68.78%	3	4	85	0.486	0.544	0.872	3	0.778	0.822	0.904	5	1102	124.2	8.54	0.634	0.835
IPI00320480.3	Sphingosine-1-phosphate lyase 1	56.69%	2	6	44	0.680	0.605	0.689	5	0.853	0.893	0.864	11	568	63.6	9.10	0.658	0.870
IPI00223047.2	Cytoskeleton-associated protein 4	91.13%	6	32	83	0.641	0.663	0.675	42	0.775	0.800	0.805	53	575	63.7	5.64	0.660	0.793
IPI00114733.2	Serpin H1	85.13%	1	21	53	0.638	0.666	0.683	33	0.809	0.847	0.875	49	417	46.5	8.82	0.662	0.844
IPI00654388.2	Leucine-rich repeat flightless-interacting protein 1	87.38%	5	16	72	0.680	0.689	0.650	39	0.893	0.877	0.833	63	729	79.2	4.82	0.673	0.868
IPI00308938.6	Calpain-2 catalytic subunit	68.86%	1	14	50	0.748	0.730	0.747	17	0.819	0.793	0.829	18	700	79.8	4.96	0.742	0.814
IPI00751474.1	Plasma membrane calcium ATPase 4 isoform x/e	66.64%	7	5	95	0.712	0.750	0.773	12	0.772	0.887	0.856	19	1205	133.0	6.13	0.745	0.838
IPI00474959.2	Putative uncharacterized protein	45.63%	1	9	20	0.600	0.806	0.846	5	0.916	0.889	0.846	15	355	41.0	4.59	0.751	0.884
IPI00222675.3	D19Bwg1357e protein	73.92%	1	8	66	0.749	0.774	0.794	6	0.842	0.901	0.870	9	648	72.9	9.66	0.772	0.871
IPI00311453.5	putative ribosomal RNA methyltransferase NOP2	59.57%	1	11	60	0.688	0.788	0.850	13	1.273	1.303	1.277	16	794	86.9	9.19	0.775	1.284
IPI00309768.7	PDZ and LIM domain protein 1	88.07%	1	9	28	0.750	0.768	0.830	16	0.751	0.702	0.735	20	327	35.8	6.84	0.783	0.729
IPI00346834.1	Keratin, type II cytoskeletal 2 oral	79.29%	9	1	68	1.382	1.233	1.191	5	0.797	0.870	0.854	8	594	62.8	8.43	1.269	0.840
IPI00112963.1	Catenin alpha-1	84.99%	3	26	107	1.313	1.229	1.292	63	0.871	0.817	0.887	49	906	100.0	6.23	1.278	0.858
IPI00111218.1	Aldehyde dehydrogenase, mitochondrial	77.46%	1	11	45	1.343	1.307	1.297	21	0.661	0.686	0.715	28	519	56.5	7.62	1.316	0.687
IPI00318614.9	Isocitrate dehydrogenase [NADP], mitochondrial	97.12%	4	18	61	1.401	1.374	1.255	25	1.179	1.157	1.165	23	452	50.9	8.69	1.343	1.167
IPI00119115.2	V-type proton ATPase subunit E 1	83.19%	2	5	35	1.394	1.279	1.377	5	0.802	0.889	0.806	5	226	26.1	8.43	1.350	0.832
IPI00131176.1	Cytochrome c oxidase subunit 2	44.93%	1	4	7	1.414	1.465	1.200	9	1.306	1.108	1.071	11	227	26.0	4.73	1.360	1.162
IPI00229475.1	Junction plakoglobin	78.52%	1	17	74	1.447	1.320	1.361	25	0.819	0.877	0.839	18	745	81.7	6.14	1.376	0.845
IPI00313968.8	Histone-lysine N-methyltransferase setd3	66.16%	3	5	48	1.429	1.383	1.358	7	0.819	0.829	0.773	7	594	67.1	5.60	1.390	0.807
IPI00553419.3	Desmoplakin	80.06%	2	28	362	1.465	1.279	1.500	27	0.865	0.877	0.791	12	2883	332.7	6.80	1.415	0.844
IPI00751369.1	L-lactate dehydrogenase A chain isoform 2	96.68%	5	20	51	1.506	1.393	1.369	59	1.145	1.126	1.085	57	361	39.7	8.35	1.423	1.119
IPI00461514.4	Histone H2B	90.37%	21	11	27	1.442	1.475	1.353	53	1.262	1.172	1.046	58	135	14.9	10.13	1.423	1.160
IPI00229510.5	L-lactate dehydrogenase B chain	75.15%	6	4	34	1.476	1.326	1.482	4	1.194	1.185	1.208	4	334	36.5	6.05	1.428	1.196
IPI00135324.2	Solute carrier family 12 member 2	56.05%	2	23	73	1.292	1.477	1.544	33	0.875	0.705	0.621	6	1206	130.6	6.52	1.438	0.734
IPI00125899.1	Catenin beta-1	79.90%	3	12	63	1.466	1.476	1.504	11	0.738	0.785	0.800	9	781	85.4	5.86	1.482	0.774
IPI00121550.3	Sodium/potassium-transporting ATPase subunit beta-1	64.47%	1	7	23	1.484	1.554	1.421	13	0.865	0.737	0.850	3	304	35.2	8.65	1.486	0.817
IPI00222386.3	Charged multivesicular body protein 2b	60.56%	1	5	26	1.592	1.481	1.389	9	0.787	0.767	0.837	3	213	23.9	8.78	1.487	0.797
IPI00282848.1	20 kDa protein	87.85%	2	1	32	1.574	1.547	1.464	18	1.249	1.159	1.067	13	181	20.2	11.39	1.528	1.158
IPI00320188.5	Nicotinamide phosphoribosyltransferase	76.99%	1	7	40	1.588	1.552	1.532	3	1.129	1.140	1.118	6	491	55.4	7.15	1.557	1.129
IPI00553294.1	Uncharacterized protein	71.74%	9	1	13	1.681	1.862	1.285	36	1.421	1.272	1.000	35	92	9.6	9.99	1.609	1.231
IPI00331734.5	Histone H2A.Z	88.28%	5	4	17	1.568	1.746	1.548	5	1.203	1.135	1.109	5	128	13.5	10.58	1.621	1.149
IPI00319556.6	Histone H1t	70.81%	1	1	33	1.812	1.674	1.391	14	1.311	1.099	0.945	5	209	21.7	11.60	1.626	1.118
IPI00553538.3	Histone H3.1	77.21%	1	1	19	1.750	1.689	1.528	4	1.249	1.159	1.067	13	136	15.4	11.12	1.656	1.158
IPI00230113.5	Cytochrome b5	88.81%	3	5	12	1.731	1.748	1.670	12	0.906	0.848	0.844	9	134	15.2	5.07	1.716	0.866
IPI00223714.5	Histone H1.4	86.76%	8	7	52	1.912	1.805	1.443	74	1.410	1.282	0.988	74	219	22.0	11.11	1.720	1.227
IPI00331597.6	Histone H1.3	90.95%	2	3	55	1.816	1.893	1.490	93	1.389	1.228	0.972	73	221	22.1	11.03	1.733	1.196
IPI00223713.5	Histone H1.2	87.26%	1	6	49	1.810	1.882	1.525	60	1.296	1.153	0.964	47	212	21.3	11.00	1.739	1.138
IPI00227140.1	Keratin, type I cytoskeletal 14	79.75%	10	7	61	1.862	1.929	1.611	24	0.561	0.646	0.572	14	484	52.8	5.17	1.801	0.593
IPI00112947.1	Keratin, type I cytoskeletal 19	97.27%	5	28	66	1.983	2.014	1.979	86	0.709	0.719	0.739	40	403	44.5	5.39	1.992	0.722
IPI00311626.5	HMG nucleosome-binding domain-containing protein 5	57.39%	1	8	31	2.155	2.296	2.176	4	1.159	1.135	1.114	8	406	45.3	4.37	2.209	1.136
IPI00131933.1	Claudin-2	51.74%	1	3	9	2.223	2.690	1.848	17	0.878	0.883	0.885	6	230	24.5	7.93	2.254	0.882
IPI00468696.3	Keratin, type I cytoskeletal 42	84.29%	5	1	56	2.556	2.229	1.976	12	0.371	0.391	0.518	5	452	50.1	5.16	2.254	0.427
