# Supplementary material for: The Prion Protein Controls Polysialylation of Neural Cell Adhesion Molecule 1 during Cellular Morphogenesis
Source: PLoS One. 2015 Aug 19;10(8):e0133741. doi: 10.1371/journal.pone.0133741 (PMC4546001; doi:10.1371/journal.pone.0133741)
Supplement: S3 Table — (PDF) [file pone.0133741.s005.pdf]

S3 Table: Top 200 proteins exhibiting most pronounced differences in expression levels in direct comparison of 48 hours TGFB1 treated stable PrP kd v wt cells (extracted from dataset II)

Accession	Original database description	Modified description	Coverage	# Proteins	# Unique Peptides	# Peptides	# PSMs	TMT Ratios 126/131	Count	Variability [%]	127/131	Count	Variability [%]	128/131	Count	Variability [%]	129/131	Count	Variability [%]	130/131	Count	Variability [%]	# Peptides Sequest	Mascot	# PSM Sequest	Mascot	Coverage Sequest	Mascot	Score Sequest	Mascot	# AAs	MW [kDa]	Averages pI	stable kd v. wt
IPI00122257.1	5'-nucleotidase	5'-nucleotidase	71.01%	2	7	43	175	0.374	7	37.4	0.972	7	6.8	0.373	7	40.4	0.855	7	15.7	0.375	7	31.5	10	12	17	29	30.90%	34.38%	32.24	91.71	576	63.8	6.64	0.374
IPI00468696.3	Keratin, type I cytoskeletal 42	Keratin, type I cytoskeletal 42	84.29%	5	1	56	468	0.371	5	151.1	1.069	5	24.7	0.391	5	94.7	1.086	5	7.6	0.518	5	50.6	11	14	39	52	24.78%	36.50%	38	114.54	452	50.1	5.16	0.427
IPI00406377.3	Keratin, type II cytoskeletal 7	Keratin, type II cytoskeletal 7	95.19%	8	23	75	1031	0.538	54	67	1.007	58	13.7	0.572	54	64.2	0.967	58	14.6	0.594	52	62.2	29	34	96	122	57.77%	63.68%	186.75	419.11	457	50.7	5.87	0.568
IPI00111286.1	Cysteine-rich with EGF-like domain protein 2	Cysteine-rich with EGF-like domain protein 2	82.29%	1	2	20	97	0.816	4	23.3	1.079	4	21	0.359	4	64.8	0.94	4	18.1	0.586	4	67.2	4	5	9	10	22.29%	25.14%	14.24	34.79	350	38.2	4.58	0.587
IPI00227140.1	Keratin, type I cytoskeletal 14	Keratin, type I cytoskeletal 14	79.75%	10	7	61	410	0.561	14	58.8	1.066	14	17.5	0.646	14	51.2	0.927	14	18.9	0.572	14	75.8	21	19	38	52	45.04%	48.55%	43.17	216.77	484	52.8	5.17	0.593
IPI00608020.4	Ferritin light chain 1	Ferritin light chain 1	85.25%	3	5	21	301	0.592	26	61.7	1.066	27	24.8	0.607	27	42.1	0.963	27	26.8	0.596	26	50	10	6	59	51	56.28%	41.53%	125.45	355.17	183	20.8	6	0.598
IPI00459639.3	FERM domain-containing protein 8	FERM domain-containing protein 8	52.79%	2	2	26	147	0.581	3	50.3	0.754	3	0.4	0.449	3	115.3	0.941	4	7.8	0.928	4	10.3	6	11	8	22	11.80%	33.69%	14.43	24.81	466	51.8	6.44	0.653
IPI00111218.1	Aldehyde dehydrogenase, mitochondrial	Aldehyde dehydrogenase, mitochondrial	77.46%	1	11	45	367	0.661	29	26.5	0.997	29	7.5	0.686	29	25.7	0.975	29	11	0.715	28	22.5	16	12	54	42	33.72%	26.97%	115.41	340.39	519	56.5	7.62	0.687
IPI00153129.1	UDP-glucose 4-epimerase	UDP-glucose 4-epimerase	58.50%	1	4	20	113	0.562	4	40.5	1.002	5	60.4	0.763	5	14.3	1.073	5	22.4	0.748	5	59	3	11	7	21	12.39%	43.80%	23.69	104.7	347	38.2	6.74	0.691
IPI00124513.1	LIM and cysteine-rich domains protein 1	LIM and cysteine-rich domains protein 1	73.70%	1	5	32	158	0.671	9	17.2	0.992	9	9.1	0.696	9	20.4	0.996	9	6.6	0.714	9	14.8	14	13	24	34	37.53%	37.26%	45.43	149.41	365	41	7.93	0.694
IPI00336281.2	Uncharacterized protein	Uncharacterized protein	84.06%	1	1	159	841	0.51	3	27.7	0.76	3	40.9	0.764	3	26.1	0.811	3	16.7	0.879	2	6.8	48	56	75	148	34.16%	42.30%	80.92	41	1487	167.2	5.38	0.718
IPI00407413.2	SCAN domain containing 3	SCAN domain containing 3	53.84%	4	1	57	229	0.734	6	2.6	0.972	6	1.8	0.679	6	14.6	0.963	6	2.6	0.749	6	5.8	12	14	25	25	15.08%	26.19%	23.93	46.07	756	84.2	8.44	0.721
IPI00112947.1	Keratin, type I cytoskeletal 19	Keratin, type I cytoskeletal 19	97.27%	5	28	66	1088	0.709	40	24.3	0.964	40	7.4	0.719	40	26.4	0.957	40	8.6	0.739	40	30.2	28	30	74	85	74.94%	78.66%	122.45	292.31	403	44.5	5.39	0.722
IPI00309768.7	PDZ and LIM domain protein 1	PDZ and LIM domain protein 1	88.07%	1	9	28	428	0.751	19	24.6	0.955	23	15.7	0.702	21	31.5	0.942	23	18.2	0.735	20	32.7	9	8	57	103	33.03%	36.70%	116.95	429.28	327	35.8	6.84	0.729
IPI00135324.2	Solute carrier family 12 member 2	Solute carrier family 12 member 2	56.05%	2	23	73	414	0.875	6	35.6	0.865	6	26.3	0.705	6	69.6	0.914	6	25	0.621	6	69.2	18	25	29	43	22.14%	25.87%	38.19	55.39	1206	130.6	6.52	0.734
IPI00468924.5	Calcium-binding mitochondrial carrier protein SCaMC-1	Calcium-binding mitochondrial carrier protein SCaMC-1	81.05%	1	13	50	306	0.72	9	8.2	0.964	9	12.3	0.741	9	16.9	0.953	9	4.3	0.741	9	9.6	14	18	25	40	40.63%	49.89%	31.39	37.72	475	52.9	7.43	0.734
IPI00230665.3	Neural cell adhesion molecule 1 isoform 3	Neural cell adhesion molecule 1 isoform 3	73.27%	4	14	84	731	0.762	58	21.5	0.986	59	17.5	0.757	58	20.9	0.944	59	11.8	0.751	57	20.9	32	35	110	107	34.35%	42.78%	218.89	247.08	1115	119.2	4.82	0.757
IPI00281011.7	MARCKS-related protein	MARCKS-related protein	91.50%	1	8	13	297	0.736	24	14.8	0.972	28	14.6	0.789	26	16.7	0.965	25	9.4	0.756	26	12.9	10	8	54	49	87.50%	64.50%	139.55	442.63	200	20.2	4.61	0.760
IPI00125653.2	Perilipin-2	Perilipin-2	83.06%	3	5	39	184	0.776	8	18	0.948	9	5	0.793	9	28	0.932	9	14.6	0.744	9	14.2	9	19	20	47	23.29%	56.71%	31.75	34.17	425	46.6	6.87	0.771
IPI00125899.1	Catenin beta-1	Catenin beta-1	79.90%	3	12	63	327	0.738	9	39.1	0.972	9	13.4	0.785	9	24.1	0.944	9	11.4	0.8	9	34.9	20	20	37	42	32.91%	33.29%	43.43	55.96	781	85.4	5.86	0.774
IPI00230141.2	Isoform Short of Long-chain-fatty-acid--CoA ligase 4	Long-chain-fatty-acid-CoA ligase 4	63.58%	2	8	44	192	0.806	6	27.1	0.943	6	14.7	0.786	6	22.8	0.814	6	33.5	0.74	6	48.7	17	8	23	22	29.10%	13.13%	28.37	82.18	670	74.3	8	0.777
IPI00377732.3	Protein FAM91A1	FAM91A1	57.59%	1	3	63	269	0.837	5	29.4	0.93	6	42.2	0.823	6	31.5	0.892	6	22.4	0.687	4	71.8	14	17	29	33	19.59%	20.55%	32.52	48.16	837	93.4	6.35	0.782
IPI00320420.3	Clusterin	Clusterin	62.28%	1	10	39	249	0.741	22	40.4	0.928	22	11.1	0.818	22	50.7	0.954	22	11.4	0.815	22	39.7	15	17	35	51	30.58%	44.42%	60.43	148.64	448	51.6	5.67	0.791
IPI00957112.1	Calpain small subunit 1	Calpain small subunit 1	82.46%	2	5	23	178	0.861	12	16.5	1.027	12	17.6	0.827	12	14.2	0.914	12	11.5	0.691	12	23.1	6	9	19	30	45.90%	47.76%	42.42	299.15	268	28.4	5.63	0.793
IPI00223047.2	Cytoskeleton-associated protein 4	Cytoskeleton-associated protein 4	91.13%	6	32	83	753	0.775	53	25.5	1.035	53	18.5	0.8	53	20.4	0.958	53	13	0.805	53	22.1	38	37	101	145	65.22%	61.22%	197.88	565.22	575	63.7	5.64	0.793
IPI00387370.1	Isoform AGX2 of UDP-N-acetylhexosamine pyrophosphorylase	UDP-N-acetylhexosamine pyrophosphorylase	58.81%	5	9	36	214	0.773	9	14.3	0.999	9	2.5	0.808	9	9.1	1.025	9	7.7	0.801	9	14.1	11	11	32	38	22.80%	31.99%	32.62	56.6	522	58.6	6.49	0.794
IPI00222386.3	Charged multivesicular body protein 2b	Charged multivesicular body protein 2b	60.56%	1	5	26	162	0.787	3	11.5	0.999	3	4	0.767	3	10.2	1.003	3	13	0.837	3	0.5	11		24		37.56%		26.37		213	23.9	8.78	0.797
IPI00130589.8	Superoxide dismutase [Cu-Zn]	Superoxide dismutase [Cu-Zn]	70.13%	2	10	17	245	0.783	23	20.9	0.962	23	24.3	0.803	23	13.9	1.053	23	16.4	0.818	23	15.6	11	12	36	34	50.00%	63.64%	60.8	150.05	154	15.9	6.51	0.801
IPI00118787.1	V-type proton ATPase subunit D	V-type proton ATPase subunit D	69.23%	3	4	22	77	0.721	3	12.4	1	3	4.2	1.014	3	12	1.002	3	11.4	0.678	3	76.4	7		10		32.39%		15.29		247	28.4	9.45	0.804
