# Supplementary material for: The Prion Protein Controls Polysialylation of Neural Cell Adhesion Molecule 1 during Cellular Morphogenesis
Source: PLoS One. 2015 Aug 19;10(8):e0133741. doi: 10.1371/journal.pone.0133741 (PMC4546001; doi:10.1371/journal.pone.0133741)
Supplement: S5 Table — (PDF) [file pone.0133741.s007.pdf]

S5 Table. Similarities amongst prior independent observations of PrP- and PSA-NCAM-related phenotypes

Phenotype		PrP-related observations	PSA-NCAM related observations
1. circadian rhythm	a.	ko mice exhibit altered circadian rhythm under continuous darkness [1]	critical for intact free-running circadian rhythmicity under continuous darkness [3,4]
	b.	diurnal fluctuations of PrP mRNA [2]	diurnal fluctuations of PSA-NCAM levels [5]
2. mossy fiber pathfinding		ko mice exhibit pathfinding defect of infrapyrimidal bundle, causing it to run underneath pyramidal cell layer [6]	NCAM ko mice and ST8SIA2 ko mice exhibit pathfinding defect of infrapyrimidal bundle, causing it to run underneath pyramidal cell layer [7, 8, 9]
3. neurogenesis		observed within adult subventricular zone in neuroblasts; its presence promotes their differentiation into mature neurons [10]	marker of adult neurogenesis research; observed within adult subventricular zone in neuroblasts [11,12,13]
4. LTP and voltage-gated ion channels	a.	ko mice exhibit deficits in LTP [14,15,16]	promotes LTP [19]
	b.	promotes AMPARs [17]	promotes AMPARs [20]
	c.	restricts activity of NMDARs [18]	restricts activity of NMDARs [21]
5. hematopoietic stem cells (HSCs)	a.	not found on totipotent embryonic stem cells (ESCs) but expressed on HSCs [22]	not found on totipotent embryonic stem cells but expressed on HSCs [26]
	b.	ko mice exhibit impaired HSC self renewal [23]	ST8SIA4-ko mice exhibit 30% lower levels of thymocytes [27]
	c.	expression is high in monocytes dendritic cells (DCs), T- and B-lymphocytes and natural killer cells (NK) [24]	expression is high in monocytes and NK cells;
	d.	neuropilin is a candidate PrP interactor [25]	as monocytes differentiate into DCs, the substrate for PSA attachment shifts from NCAM to neuropilin [28,29]
6. myelin repair	a.	expressed on axon and non-myelinating Schwann cells [30]	expressed on axons and non-myelinating Schwann cells [31,32];
	b.	ko causes peripheral neuropathy characterized by a myelin maintenance defect [30]	implicated in regeneration of lesioned or chemically demyelinated peripheral nerves [32,33,34,35]
7. dentin abnormalities		ko mice exhibit dentin structure defects [36]	NCAM levels in progenic dental pulp correlate with number of dentin producing active odontoblasts [37]
8. resistance to Brucella		may contribute to the cellular uptake of bacteria belonging to the *Brucella* family [38,39]	adherence of *Brucella* bacteria to human epithelial cells is mediated by sialic acid residues [40,41]

References to S5 Table

1. Tobler I, Gaus SE, Deboer T, Achermann P, Fischer M, Rulicke T, et al. Altered circadian activity rhythms and sleep in mice devoid of prion protein. Nature 1996; 380: 639-642.
2. Cagampang FR, Whatley SA, Mitchell AL, Powell JF, Campbell IC, Coen CW Circadian regulation of prion protein messenger RNA in the rat forebrain: a widespread and synchronous rhythm. Neuroscience 1999; 91: 1201-1204.
3. Shen H, Watanabe M, Tomasiewicz H, Glass JD Genetic deletions of NCAM and PSA impair circadian function in the mouse. Physiol Behav 2001; 73: 185-193.
4. Shen H, Watanabe M, Tomasiewicz H, Rutishauser U, Magnuson T, Glass JD Role of neural cell adhesion molecule and polysialic acid in mouse circadian clock function. J Neurosci 1997; 17: 5221-5229.
5. Glass JD, Watanabe M, Fedorkova L, Shen H, Ungers G, Rutishauser U Dynamic regulation of polysialylated neural cell adhesion molecule in the suprachiasmatic nucleus. Neuroscience 2003; 117: 203-211.
6. Colling SB, Khana M, Collinge J, Jefferys JG Mossy fibre reorganization in the hippocampus of prion protein null mice. Brain Res 1997; 755: 28-35.
7. Seki T, Rutishauser U Removal of polysialic acid-neural cell adhesion molecule induces aberrant mossy fiber innervation and ectopic synaptogenesis in the hippocampus. J Neurosci 1998; 18: 3757-3766.
8. Angata K, Long JM, Bukalo O, Lee W, Dityatev A, Wynshaw-Boris A, et al. Sialyltransferase ST8Sia-II assembles a subset of polysialic acid that directs hippocampal axonal targeting and promotes fear behavior. J Biol Chem 2004; 279: 32603-32613.
9. Eckhardt M, Bukalo O, Chazal G, Wang L, Goridis C, Schachner M, et al. Mice deficient in the polysialyltransferase ST8SiaIV/PST-1 allow discrimination of the roles of neural cell adhesion molecule protein and polysialic acid in neural development and synaptic plasticity. J Neurosci 2000; 20: 5234-5244.
10. Steele AD, Emsley JG, Ozdinler PH, Lindquist S, Macklis JD Prion protein (PrPc) positively regulates neural precursor proliferation during developmental and adult mammalian neurogenesis. Proc Natl Acad Sci USA 2006; 103: 3416-3421.

11. Doetsch F, Garcia-Verdugo JM, Alvarez-Buylla A Cellular composition and three-dimensional organization of the subventricular germinal zone in the adult mammalian brain. J Neurosci 1997; 17: 5046-5061.
12. Bonfanti L PSA-NCAM in mammalian structural plasticity and neurogenesis. Prog Neurobiol 2006; 80: 129-164.
13. Gascon E, Vutskits L, Kiss JZ The role of PSA-NCAM in adult neurogenesis. Adv Exp Med Biol 2010; 663: 127-136.
14. Collinge J, Whittington MA, Sidle KC, Smith CJ, Palmer MS, Clarke AR, et al. Prion protein is necessary for normal synaptic function. Nature 1994; 370: 295-297.
15. Curtis J, Errington M, Bliss T, Voss K, MacLeod N Age-dependent loss of PTP and LTP in the hippocampus of PrP-null mice. Neurobiol Dis 2003; 13: 55-62.
16. Rangel A, Madronal N, Gruart A, Gavin R, Llorens F, Sumoy L, et al. Regulation of GABA(A) and glutamate receptor expression, synaptic facilitation and long-term potentiation in the hippocampus of prion mutant mice. PloS One 2009; 4: e7592.
17. Watt NT, Taylor DR, Kerrigan TL, Griffiths HH, Rushworth JV, Whitehouse IJ, et al. Prion protein facilitates uptake of zinc into neuronal cells. Nat Commun 2012; 3: 1134.
18. Khosravani H, Zhang Y, Tsutsui S, Hameed S, Altier C, Hamid J, et al. Prion protein attenuates excitotoxicity by inhibiting NMDA receptors. J Cell Biol 2008; 181: 551-565.
19. Senkov O, Sun M, Weinhold B, Gerardy-Schahn R, Schachner M, Dityatev A Polysialylated neural cell adhesion molecule is involved in induction of long-term potentiation and memory acquisition and consolidation in a fear-conditioning paradigm. J Neurosci 2006; 26: 10888-109898.
20. Senkov O, Tikhobrazova O, Dityatev A PSA-NCAM: synaptic functions mediated by its interactions with proteoglycans and glutamate receptors. Int J Biochem Cell Biol 2012; 44: 591-595.
21. Kochlamazashvili G, Senkov O, Grebenyuk S, Robinson C, Xiao MF, Stummeyer K, et al. Neural cell adhesion molecule-associated polysialic acid regulates synaptic plasticity and learning by restraining the signaling through GluN2B-containing NMDA receptors. J Neurosci 2010; 30: 4171-4183.
22. Dodelet VC, Cashman NR Prion protein expression in human leukocyte differentiation. Blood 1998; 91: 1556-1561.

23. Zhang CC, Steele AD, Lindquist S, Lodish HF Prion protein is expressed on long-term repopulating hematopoietic stem cells and is important for their self-renewal. Proc Natl Acad Sci U S A 2006; 103: 2184-2189.
24. Isaacs JD, Jackson GS, Altmann DM The role of the cellular prion protein in the immune system. Clin Exp Immunol 2006; 146: 1-8.
25. Watts JC, Huo H, Bai Y, Ehsani S, Jeon AH, Shi T, et al. Interactome analyses identify ties of PrP and its mammalian paralogs to oligomannosidic N-glycans and endoplasmic reticulum-derived chaperones. PLoS Pathog 2009; 5: e1000608.
26. Drake PM, Nathan JK, Stock CM, Chang PV, Muench MO, Nakata D, et al. Polysialic acid, a glycan with highly restricted expression, is found on human and murine leukocytes and modulates immune responses. J Immunol 2008; 181: 6850-6858.
27. Drake PM, Stock CM, Nathan JK, Gip P, Golden KP, Weinhold B, et al. Polysialic acid governs T-cell development by regulating progenitor access to the thymus. Proc Natl Acad Sci USA 2009; 106: 11995-12000.
28. Curreli S, Arany Z, Gerardy-Schahn R, Mann D, Stamatos NM Polysialylated neuropilin-2 is expressed on the surface of human dendritic cells and modulates dendritic cell-T lymphocyte interactions. J Biol Chem 2007; 282: 30346-30356.
29. Rey-Gallardo A, Delgado-Martin C, Gerardy-Schahn R, Rodriguez-Fernandez JL, Vega MA Polysialic acid is required for neuropilin-2a/b-mediated control of CCL21-driven chemotaxis of mature dendritic cells and for their migration in vivo. Glycobiology 2011; 21: 655-662.
30. Bremer J, Baumann F, Tiberi C, Wessig C, Fischer H, Schwarz P, et al. Axonal prion protein is required for peripheral myelin maintenance. Nat Neurosci 2010; 13: 310-318.
31. Martini R, Schachner M Immunoelectron microscopic localization of neural cell adhesion molecules (L1, N-CAM, and MAG) and their shared carbohydrate epitope and myelin basic protein in developing sciatic nerve. J Cell Biol 1986; 103: 2439-2448.
32. Nait Oumesmar B, Vignais L, Duhamel-Clerin E, Avellana-Adalid V, Rougon G, Baron-Van Evercooren A Expression of the highly polysialylated neural cell adhesion molecule during postnatal myelination and following chemically induced demyelination of the adult mouse spinal cord. Eur J Neurosci 1995; 7: 480-491.
33. Martini R, Schachner M Immunoelectron microscopic localization of neural cell adhesion molecules (L1, N-CAM, and myelin-associated glycoprotein) in regenerating adult mouse sciatic nerve. J Cell Biol 1988; 106: 1735-1746.

34. Daniloff JK, Levi G, Grumet M, Rieger F, Edelman GM Altered expression of neuronal cell adhesion molecules induced by nerve injury and repair. J Cell Biol 1986; 103: 929-945.
35. Covault J, Merlie JP, Goridis C, Sanes JR Molecular forms of N-CAM and its RNA in developing and denervated skeletal muscle. J Cell Biol 1986; 102: 731-739.
36. Schneider K, Korkmaz Y, Addicks K, Lang H, Raab WH Prion protein (PrP) in human teeth: an unprecedented pointer to PrP's function. J Endod 2007; 33: 110-113.
37. Ustiashvili M, Kordzaia D, Mamaladze M, Jangavadze M, Sanodze L Investigation of functional activity human dental pulp stem cells at acute and chronic pulpitis. Georgian Med News 2014: 19-24.
38. Watarai M, Kim S, Erdenebaatar J, Makino S, Horiuchi M, Shirahata T, et al. Cellular prion protein promotes Brucella infection into macrophages. J Exp Med 2003; 198: 5-17.
39. Nakato G, Hase K, Suzuki M, Kimura M, Ato M, Hanazato M, et al. Cutting Edge: Brucella abortus exploits a cellular prion protein on intestinal M cells as an invasive receptor. J Immunol 2012; 189: 1540-1544.
40. Castaneda-Roldan EI, Avelino-Flores F, Dall'Agnol M, Freer E, Cedillo L, Dornand J, et al. Adherence of Brucella to human epithelial cells and macrophages is mediated by sialic acid residues. Cell Microbiol 2004; 6: 435-445.
41. del CR-GR, Castaneda-Roldan EI, Giono-Cerezo S, Giron JA Brucella sp. bind to sialic acid residues on human and animal red blood cells. FEMS Microbiol Lett 2002; 213: 219-224.
